# Evaluating Effectiveness of mHealth Interventions for Rehabilitation in Patients With Head and Neck Cancer: Systematic Review and Meta-Analysis of Randomized Controlled Trials

**DOI:** 10.2196/78109

**Published:** 2026-04-30

**Authors:** Yang Xiao, Qiuyu Zhou, Feiruo Hong, Yuwei Lin, Enhong Li, Xuefen Yu

**Affiliations:** 1 School of Medicine, Zhejiang University, Hangzhou, Zhejiang, China; 2 Stomatology Hospital, School of Stomatology, Zhejiang University School of Medicine, Zhejiang Provincial Clinical Research Center for Oral Diseases, Zhejiang Key Laboratory of Oral Biomedical, Hangzhou, Zhejiang, 310000, China, 86 13067987798

**Keywords:** mobile health, head and neck cancer, rehabilitation, quality of life, depression, pain, systematic review, meta-analysis

## Abstract

**Background:**

Patients with head and neck cancer (HNC) frequently experience functional impairments and psychological distress following surgery or radiotherapy. While mobile health (mHealth) interventions are increasingly integrated into clinical care to support patient self-management and home-based recovery, evidence of their effectiveness in HNC remains inconsistent.

**Objective:**

This study aims to evaluate the effectiveness of mHealth interventions on quality of life (QoL), psychological symptoms, physical symptoms, and functional recovery among HNC survivors.

**Methods:**

This systematic review and meta-analysis followed the PRISMA (Preferred Reporting Items for Systematic Reviews and Meta-Analyses) guidelines. Overall, 12 electronic databases were searched for randomized controlled trials published up to December 1, 2025. Two reviewers independently performed study selection, data extraction, and risk of bias assessment using the Cochrane Risk of Bias 2.0 tool. Certainty of evidence was evaluated using the GRADE (Grading of Recommendations, Assessment, Development, and Evaluation) approach. Pooled effects were calculated as standardized mean differences (SMDs) with 95% CIs. The primary outcome was QoL; secondary outcomes included anxiety, depression, fatigue, pain, swallowing function, and maximal interincisal opening (MIO).

**Results:**

A total of 26 randomized controlled trials involving 2385 participants were included. mHealth interventions significantly improved QoL (SMD 0.64, 95% CI 0.41-0.88; *P*<.001), anxiety (SMD −0.75, 95% CI −1.42 to −0.08; *P*=.03), depression (SMD −0.89, 95% CI −1.37 to −0.40; *P*<.001), and fatigue (SMD −0.85, 95% CI −1.19 to −0.51; *P*<.001). Pain showed a small reduction (SMD −0.37, 95% CI −0.49 to −0.24; *P*<.001). However, the improvement in swallowing function reached only borderline significance (SMD 0.66, 95% CI 0.28-1.04; *P*=.04), suggesting that current evidence for this outcome is fragile. No significant effect was found for MIO (*P*=.68). Subgroup analysis revealed that interventions featuring home practice support, self-monitoring, and shorter durations (<3 months) yielded stronger clinical effects. The overall certainty of evidence ranged from low to very low.

**Conclusions:**

mHealth interventions effectively enhance QoL and alleviate psychosocial distress in patients. However, evidence for improving swallowing function and MIO remains insufficient. Future research should prioritize standardized protocols and high-quality trials to validate long-term clinical value.

## Introduction

Head and neck cancer (HNC) is an umbrella term for tumors that arise in the oral cavity, oropharynx, larynx, hypopharynx, and paranasal sinuses [1]. As the seventh most common cancer globally, HNC accounted for approximately 4.7% of new cases and 4.9% of cancer-related deaths in 2022 [1-3]. Treatment for HNC typically involves surgery, radiotherapy, and systemic therapies such as chemotherapy, targeted therapy, or immunotherapy [4]. Despite advancements in these modalities, patients frequently experience severe treatment-related adverse effects. Physical impairments, including dysphagia, trismus, fatigue, and pain, frequently co-occur with psychological distress such as anxiety and depression, severely compromising quality of life (QoL) [5,6]. These symptoms can persist long-term, representing a significant burden to comprehensive rehabilitation. Studies have shown that HNC survivors report lower QoL than survivors of other cancer types [7,8].

In oncology, “rehabilitation” refers to interventions that aim to restore or maintain physical function, manage treatment-related symptoms, and improve psychological well-being during the recovery phase [9]. This stage is essential for enhancing the long-term QoL for survivors. Traditional rehabilitation models mainly rely on face-to-face consultations, which face challenges such as uneven resource distribution, limited accessibility, and low patient adherence [9,10]. Geographical barriers often prevent patients in remote areas from receiving timely, high-quality care, leaving adverse reactions poorly managed. Furthermore, time constraints in clinical settings make it difficult for physicians to capture a comprehensive record of patients’ daily symptoms. After discharge, the lack of continuous and personalized support often leads to delayed symptom management and increased complications.

Mobile health (mHealth) offers a promising solution to chronic disease management and cancer rehabilitation [11,12]. It encompasses both hardware, such as smartphones, tablets, and wearable activity tracking or sensor devices, and software, including digital health platforms and mobile apps (eg, WeChat mini-programs) [13]. By using wireless connectivity, these tools support real-time symptom monitoring, personalized rehabilitation exercises, and enhanced information accessibility [14,15]. mHealth technologies streamline patient-provider communication, promote home-based self-management, and enhance overall health outcomes. Furthermore, these applications enable patients to track their health status in real time while fostering social and psychological support through connectivity with peers and health care professionals [13].

Although mHealth shows potential, existing evidence remains inconsistent [13,16-24]. Meta-analyses by Buneviciene et al [17] and Zhang et al [24] support the effectiveness of mHealth in improving QoL and alleviating anxiety or depression in general cancer populations. However, these findings primarily derive from mixed-cancer populations. Some reviews indicate that current mHealth interventions for HNC often involve small samples and nonrandomized designs. For instance, Calver et al [18] found insufficient data to confirm the effectiveness of psychological interventions on QoL. Additionally, high heterogeneity in intervention formats and durations persists, with a notable lack of high-quality randomized controlled trials (RCTs) targeting specific outcomes such as swallowing function [13].

Therefore, based on these inconsistent findings and the recent increase in original research, this meta-analysis aims to assess the effectiveness of mHealth interventions in improving QoL, anxiety, depression, fatigue, pain, swallowing function, and maximal interincisal opening (MIO) in patients with HNC. Furthermore, we explored the effectiveness of different interventions, such as intervention delivery format and intervention durations. This study will provide evidence for the future development and clinical implementation of mHealth-based rehabilitation for patients with HNC.

## Methods

### Ethical Considerations

This article is a systematic review and meta-analysis. It does not contain any studies with human participants or animals performed by any of the authors.

### Design

The review protocol was registered with PROSPERO (CRD42024559825). We followed the PRISMA (Preferred Reporting Items for Systematic Reviews and Meta-Analyses) reporting guidelines (Checklist 1) [25].

### Data Sources and Search Strategies

We searched 12 electronic databases from their inception to December 1, 2025. These included PubMed, Embase, Cochrane Central Register of Controlled Trials, Web of Science, PsycInfo, CINAHL, Scopus, Google Scholar, China National Knowledge Infrastructure, Chinese Biomedical Literature Database, Weipu (VIP), and Wanfang Data. Search terms included Medical Subject Headings and keywords such as “head and neck cancer,” “mHealth,” “digital health,” and “RCT.” We also manually searched the reference lists of relevant articles and screened gray literature, including ClinicalTrials.gov and the World Health Organization International Clinical Trials Registry Platform. Detailed search strategies for each database are provided in Multimedia Appendix 1.

### Eligibility Criteria

We used the PICOS (participants, intervention, comparison, outcome, and study design) framework [26] to define our inclusion criteria: (1) participants: patients diagnosed with HNC of any age or gender after surgery or radiotherapy; (2) interventions: mHealth-based tools, such as smartphone apps, WeChat mini-program, wearable devices, or digital health platforms; (3) comparison: usual care, no intervention, or non-mHealth interventions; (4) outcomes: reported at least one targeted outcome, including pain, anxiety, depression, fatigue, QoL, swallowing function, MIO, adherence, self-efficacy, or perceived stress (mean and SD); (5) study design: RCTs; and (6) language: published in English or Chinese.

The exclusion criteria were as follows: (1) studies focused only on diagnosis, screening, or application development without evaluating clinical or patient-reported outcomes; (2) study types such as case reports, reviews, conference abstracts, or qualitative studies; (3) non-mHealth interventions (eg, telephone-only or face-to-face only); and (4) studies with incomplete data or results that could not be extracted.

To accurately evaluate the effectiveness of these interventions, this study adopts the following definition of key concepts. QoL refers to a multidimensional construct covering physical, psychological, and social functions [27]. Swallowing function is a core survival indicator that is often affected by chemoradiotherapy [28]. MIO serves as the key parameter for evaluating trismus after treatment [29]. Regarding mHealth functions, self-monitoring refers to patients actively recording their own symptoms [30]. Home practice support involves providing tailored rehabilitation guidance at home through digital media [31]. Telemedical support emphasizes bidirectional interaction or real-time feedback between patients and health care providers rather than the use of a simple tool [32].

### Study Selection

We used EndNote X21 (Clarivate) software to manage records and deduplication. Two reviewers (YX and QZ) independently screened titles and abstracts. Then, they evaluated the full text of potentially eligible studies. Any disagreements were resolved by a third reviewer (XY). We used Cohen κ to measure the agreement between reviewers, following standard categories: ≤0 (poor), 0.01‐0.20 (slight), 0.21‐0.40 (fair), 0.41‐0.60 (moderate), 0.61‐0.80 (substantial), and 0.81‐1.00 (almost perfect agreement) [33].

### Data Extraction

Two independent authors (YX and QZ) extracted data using a standardized Microsoft Excel spreadsheet. Extracted information included first author, year, country, setting, patient characteristics, sample size, mean age, intervention and control details, duration, follow-up, outcomes, and assessment tools. For studies with multiple control groups, we selected the most appropriate comparison, such as usual care. Any discrepancies were resolved through discussion with a third author (XY).

### Risk of Bias Assessment and Quality of Evidence

Two researchers (YX and QZ) independently assessed the risk of bias in the included RCTs using the Cochrane Risk of Bias 2.0 (RoB 2.0) tool [34]. Evaluations covered 5 domains: the randomization process, deviations from intended interventions, missing outcome data, measurement of the outcome, and selection of the reported results. Each domain was rated as “low risk,” “some concerns,” or “high risk.”

The certainty of the evidence was evaluated using the GRADE (Grading of Recommendations, Assessment, Development, and Evaluation) approach [35]. Evidence from RCTs was initially classified as “high” certainty and downgraded to “moderate,” “low,” or “very low” based on 5 factors: risk of bias, inconsistency, indirectness, imprecision, and publication bias. Conversely, evidence could be upgraded for a large effect size or dose-response gradient. For both RoB 2.0 and GRADE assessments, the κ coefficient was calculated to determine interrater agreement. Any disagreements were resolved through consensus or by consulting a third author (XY).

### Statistical Analysis

Statistical analyses were performed using Review Manager (version 5.4; The Cochrane Collaboration), Stata (version 18.0; StataCorp), and R software (version 4.4.1; R Foundation for Statistical Computing). Continuous outcomes were synthesized using standardized mean differences (SMDs) with 95% CIs. For instruments where scoring directions differed, scores were inverted to ensure a uniform orientation for each outcome. Heterogeneity between studies was assessed using the Cochrane Q test (significance level at *P*<.10) and the *I*^2^ statistic. An *I*^2^ value >50% indicated significant heterogeneity. A fixed-effects model was used when heterogeneity was low; otherwise, a random-effects model was applied [36]. Statistical significance was set at *P*<.05. The magnitude of SMD was classified as small (<0.4), medium (0.4‐0.7), or large (>0.7) [37]. Additionally, 95% prediction intervals (PIs) were calculated for outcomes with significant heterogeneity [38]. These intervals estimate the range of treatment effects expected in future clinical settings, offering a more robust interpretation of the observed variance. Subgroup and sensitivity analyses were performed to explore sources of heterogeneity. Publication bias was evaluated using funnel plots and the Egger regression test [39].

## Results

### Search Outcomes

**Figure 1.**
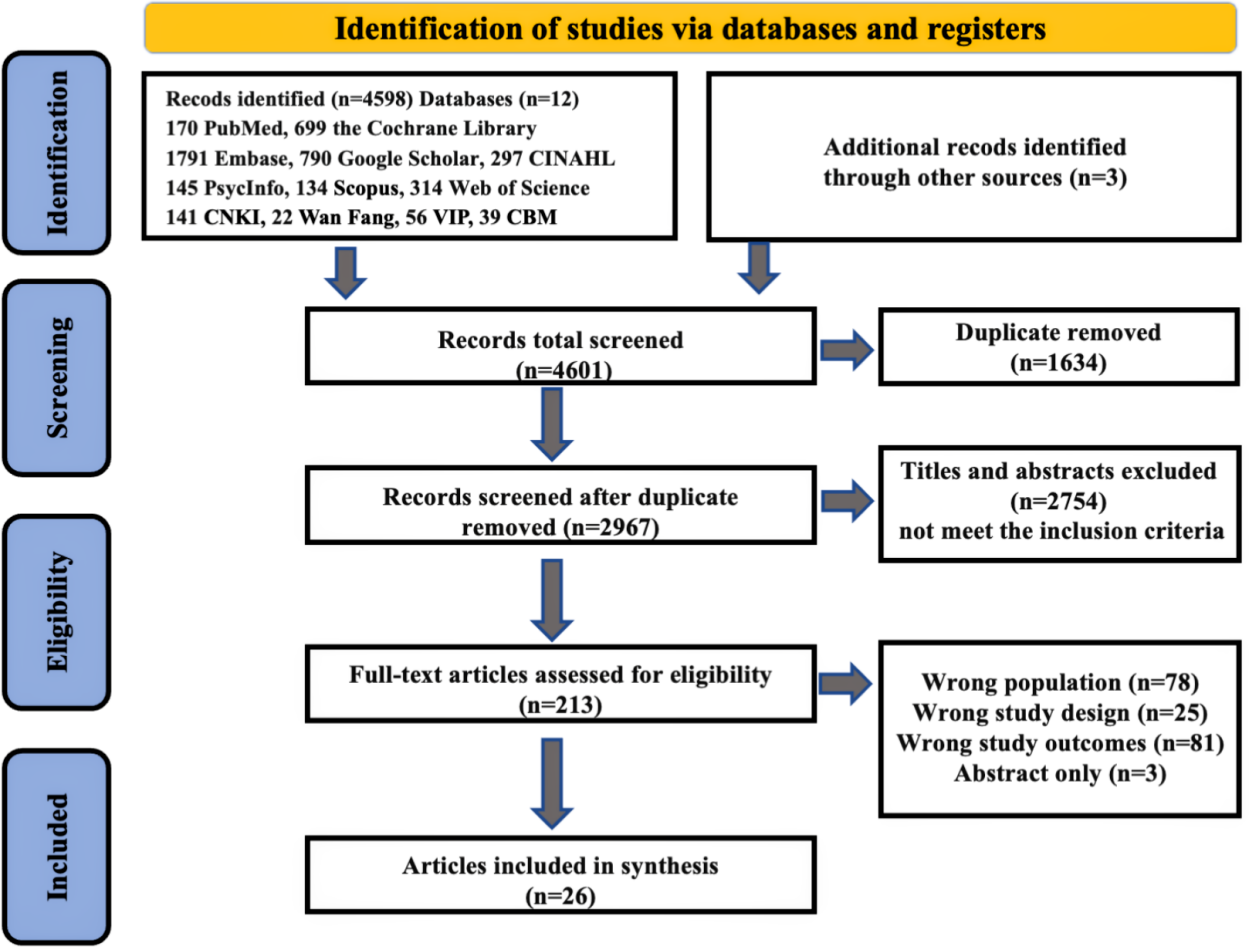
PRISMA (Preferred Reporting Items for Systematic Reviews and Meta-Analyses) flowchart of studies included in the review.

The literature search yielded 4601 records, of which 1634 were removed as duplicates. The remaining 2967 publications were evaluated at the title/abstract phase using predetermined inclusion/exclusion criteria, resulting in 2754 exclusions. Of 213 articles advancing to full-text assessment, 187 were excluded for reasons detailed in the PRISMA flowchart (Figure 1). Ultimately, 26 RCTs were included in the systematic review and meta-analysis [40-65]. The interrater reliability for study selection was substantial at the title and abstract screening stage (Cohen κ=0.85) and reached excellent agreement during the full-text review (κ=0.92).

### Study Characteristics

A total of 2385 participants were included across the 26 studies. The key characteristics of these studies are summarized in Table 1. The included trials were conducted in various countries, including China, Belgium, Canada, Korea, Germany, the Netherlands, Australia, and the United States, with publication dates ranging from 2015 to 2025. Regarding cancer subtypes, nasopharyngeal carcinoma was the most frequently studied (n=6), followed by oropharyngeal (n=3), laryngeal (n=3), oral (n=3), and thyroid cancer (n=2).

**Table 1.** General characteristics of the included studies.

Study	Country	Design	Population	Sample size, n (intervention/control)	Intervention	Control group	Intervention duration	Outcomes and outcome measures
Baudelet et al 2023 [40]	Belgium	RCT^a^	Oropharyngeal cancer	148 (49/49)	App-supported prophylactic swallowing exercises (home practice support)	Paper group/therapist group	1 month	Adherence
Cen and Yang 2019 [41]	China	RCT	Nasopharyngeal carcinoma	140 (74/75)	Health management app (follow-up reminders)	Routine health education	2 months	EORTC QLQ-H&N35^b^: fatigue, pain, swallowing function
Chen et al 2021 [42]	China	RCT	Laryngeal cancer	100 (50/50)	Mobile education app (home practice support, follow-up reminders)	Usual care	1 month	Anxiety (SAS^c^), depression (SDS^d^), quality of life (self-developed questionnaire)
Dai et al 2024 [43]	China	RCT	HNC^e^	44 (22/22)	Health enjoy system (home practice support, follow-up reminders)	Usual care	3 months	MIO^f^ (incisor measuring ruler), self-efficacy (SUPPH^g^), treatment adherence
Di et al 2017 [45]	China	RCT	Nasopharyngeal carcinoma	65 (32/33)	Multimodal smartphone app (self-monitoring and reporting, follow-up reminders)	Usual care	6 months	Quality of life, fatigue, pain (EORTC QLQ-C30^h^)
Di and Li 2018 [44]	China	RCT	Nasopharyngeal carcinoma	132 (65/67)	Medical smartphone app (self-monitoring and reporting, telemedical support, follow-up reminders)	Usual care	6 months	Quality of life (EORTC QLQ-C30)
Ducharme et al 2024 [46]	Canada	RCT	HNC	39 (24/15)	PTSD^i^ Coach game app (self-monitoring and reporting, home practice support)	Usual care	6 months	Quality of life (FACT-HN^j^), anxiety, depression (HADS)^k^
Kim and Hwang 2025 [47]	Korea	RCT	Thyroid cancer	62 (31/31)	TOP note smartwatch app (self-monitoring and reporting, home practice support, follow-up reminders)	Usual care	3 months	Perceived stress (Korea Perceived Stress Scale, NHANES version)
Lee et al 2023 [48]	Korea	RCT	HNC	40 (27/13)	ePRO-CTCAE app (self-monitoring and reporting, telemedical support)	Diary to record symptoms	2 months	Quality of life (EORTC QLQ-C30)
Liao et al 2020 [50]	China	RCT	Nasopharyngeal carcinoma	80 (40/40)	Home-based digital care platform (home practice support, follow-up reminders)	Usual care	6 months	EORTC QLQ-C30: pain, swallowing function
Liao et al 2022 [49]	China	RCT	Nasopharyngeal carcinoma	116 (58/58)	WeChat mini-program (home practice support, follow-up reminders)	Usual care	6 months	Fatigue (CRF^l^ scale), pain, swallowing function (EORTC QLQ-H&N35)
Lyu et al 2016 [51]	China	RCT	HNC	108 (57/51)	WeChat (telemedical support, follow-up reminders)	Telephone follow‐up	6 months	Patient satisfaction
Pang et al 2023 [52]	China	RCT	Oral and maxillofacial tumor	64 (33/31)	WeChat app (follow-up reminders)	Usual care	6 months	Mastication function (Modified Sato questionnaire), swallowing function (MDADI^m^)
Peng 2019 [53]	China	RCT	Oral cancer	140 (70/70)	WeChat-based interactive education platform (telemedical support, follow-up reminders)	Routine health education	6 months	UW-QOL^n^: quality of life, pain, swallowing function, anxiety; Kessler Psychological Distress Scale: depression
Pfeifer et al 2015 [54]	USA	RCT	HNC	80 (45/35)	Telemedicine for symptom management (self-monitoring and reporting, telemedical support)	Usual care	6 weeks	Quality of life (FACT-HN)
Rades et al 2022 [55]	Germany	RCT	HNC	53 (25/28)	Reminder App of skin and oral care (follow-up reminders)	Usual care	3 months	Adherence, oral mucositis
Sprave et al 2023 [56]	Germany	RCT	HNC	100 (50/50)	ePRO Monitoring System (self-monitoring and reporting, telemedical support)	Usual care	3 months	Quality of life (FACT-HN), fatigue, pain (EORTC QLQ-C30), swallowing function (ESAS^o^), anxiety, depression (HADS)
Starmer et al 2023 [57]	USA	RCT	HNC	91 (44/47)	HNC Virtual Coach with adherence and swallowing support (home practice support, self-monitoring and reporting, follow-up reminders)	Paper exercise logs	3 months	Swallowing function (MDADI), quality of life (FACT-HN)
Sun et al 2024 [58]	China	RCT	Thyroid cancer	104 (62/42)	Multitheory model-based mHealth via WeChat (follow-up reminders)	Usual care	3 months	Anxiety (PHQ-4^p^), depression (PHQ-4), quality of life (EQ-5D VAS^q^)
Van Den Brink et al 2007 [59]	Netherlands	RCT	HNC	184 (35/128)	Laptop-based eHealth support system (self-monitoring and reporting, telemedical support)	Usual care	6 weeks	Quality of life (FACT-HN)
van der Hout et al 2020 [60]	Netherlands	RCT	HNC	99 (54/45)	Oncokompas App with HRQOL^r^ and symptom monitoring (self-monitoring and reporting, follow-up reminders)	Usual care	6 months	Pain, swallowing function (EORTC QLQ-H&N35)
Wall et al 2020 [61]	Australia	RCT	Oropharyngeal cancer	79 (26/27)	SwallowIT App with remote swallowing support (home practice support, follow-up reminders)	Usual care	3 months	Swallowing function (MDADI), anxiety (HADS-A), depression (HADS-D)
Wang et al 2019 [62]	China	RCT	Oral cancer	60 (30/30)	Telephone-based (telemedical support, follow-up reminders)	Usual care	3 months	Mandibular function impairment (MFIQ^s^)
Wang et al 2025 [63]	China	RCT	Laryngeal cancer	80 (40/40)	Internet+ extended care model (home practice support, telemedical support, follow-up reminders)	Usual care	6 months	Swallowing function, quality of life, patient satisfaction (SSA^t^)
Wu et al 2025 [64]	China	RCT	HNC	108 (54/54)	Health Belief Model–based Opencare App (home practice support, follow-up reminders)	Usual care	3 months	MIO, treatment adherence, self-efficacy (HBMQ^u^), fatigue (HBMQ), pain (BPI^v^), quality of life (AQoL-6D^w^)
Xu et al 2019 [65]	China	RCT	Laryngeal cancer	60 (30/30)	Almond Doctor App (self-monitoring and reporting, home practice support, follow-up reminders)	Usual care	3 months	Anxiety (SAS), depression (SDS), quality of life (QLICP-HN^x^)

a RCT: randomized controlled trial.

b EORTC QLQ-H&N35: European Organisation for Research and Treatment of Cancer Quality of Life Questionnaire Head and Neck 35.

c SAS: Self-Rating Anxiety Scale.

d SDS: Self-Rating Depression Scale.

e HNC: Head and neck cancer.

f MIO: Maximal interincisal opening.

g SUPPH: Strategies Used by People to Promote Health.

h EORTC QLQ-C30: European Organisation for Research and Treatment of Cancer Quality of Life Questionnaire Core 30.

i PTSD: Posttraumatic stress disorder.

j FACT-HN: Functional Assessment of Cancer Therapy – Head and Neck.

k HADS: Hospital Anxiety and Depression Scale.

l CRF: Cancer-Related Fatigue scale.

m MDADI: M. D. Anderson Dysphagia Inventory.

n UW-QOL: University of Washington Quality of Life questionnaire.

o ESAS: Edmonton Symptom Assessment System.

p PHQ-4: Patient Health Questionnaire-4.

q EQ-5D VAS: EuroQol Five-Dimensional Visual Analogue Scale.

r HRQOL: Health-Related Quality of Life.

s MFIQ: Mandibular Function Impairment Questionnaire.

t SSA: Swallowing Satisfaction Assessment.

u HBMQ: Health Belief Model Questionnaire.

v BPI: Brief Pain Inventory.

w AQoL-6D: Assessment of Quality of Life 6D.

x QLICP-HN: Quality of Life Instruments for Cancer Patients – Head and Neck.

### Characteristics of Interventions and Outcome Measures

The mHealth interventions exhibited significant diversity in design and functionality. The most prevalent delivery mode was smartphone apps (n=14), which typically integrated features such as symptom monitoring, educational modules, clinical reminders, and remote follow-up (Multimedia Appendix 1). A notable subset of studies (n=6), primarily conducted in China, used WeChat-based platforms, including mini-programs and interactive education systems. Other delivery formats included smartwatch-based apps, web-based platforms, and multicomponent telemedicine models. Functional components often focused on symptom self-monitoring, home-based exercise guidance, and bidirectional clinician-patient communication. Several interventions incorporated theoretical frameworks, such as the Health Belief Model, while others used gamification or virtual coaching to improve patient engagement. Intervention durations ranged from 4 weeks to 12 months.

All assessment tools used in the included studies were validated instruments. QoL was most frequently measured using the European Organisation for Research and Treatment of Cancer Quality of Life Questionnaire Core 30 (EORTC QLQ-C30) and its head and neck-specific module (EORTC QLQ-H&N35; 10 studies), followed by the University of Washington Quality of Life questionnaire (n=3) and the Functional Assessment of Cancer Therapy–Head and Neck (n=2). For psychological outcomes, the Hospital Anxiety and Depression Scale was the most common (n=6), followed by the Self-Rating Anxiety/Depression Scale (n=3) and Patient Health Questionnaire-9/Generalized Anxiety Disorder-7 (n=4). Fatigue and pain were primarily assessed through subscales of the EORTC tools. Swallowing function was evaluated using the M. D. Anderson Dysphagia Inventory (n=3) or EORTC QLQ-H&N35 (n=2). Additional outcomes included MIO and self-efficacy (using the Strategies Used by People to Promote Health or the Health Belief Model Questionnaire, and the Mandibular Function Impairment Questionnaire).

### Risk of Bias

**Figure 2.**
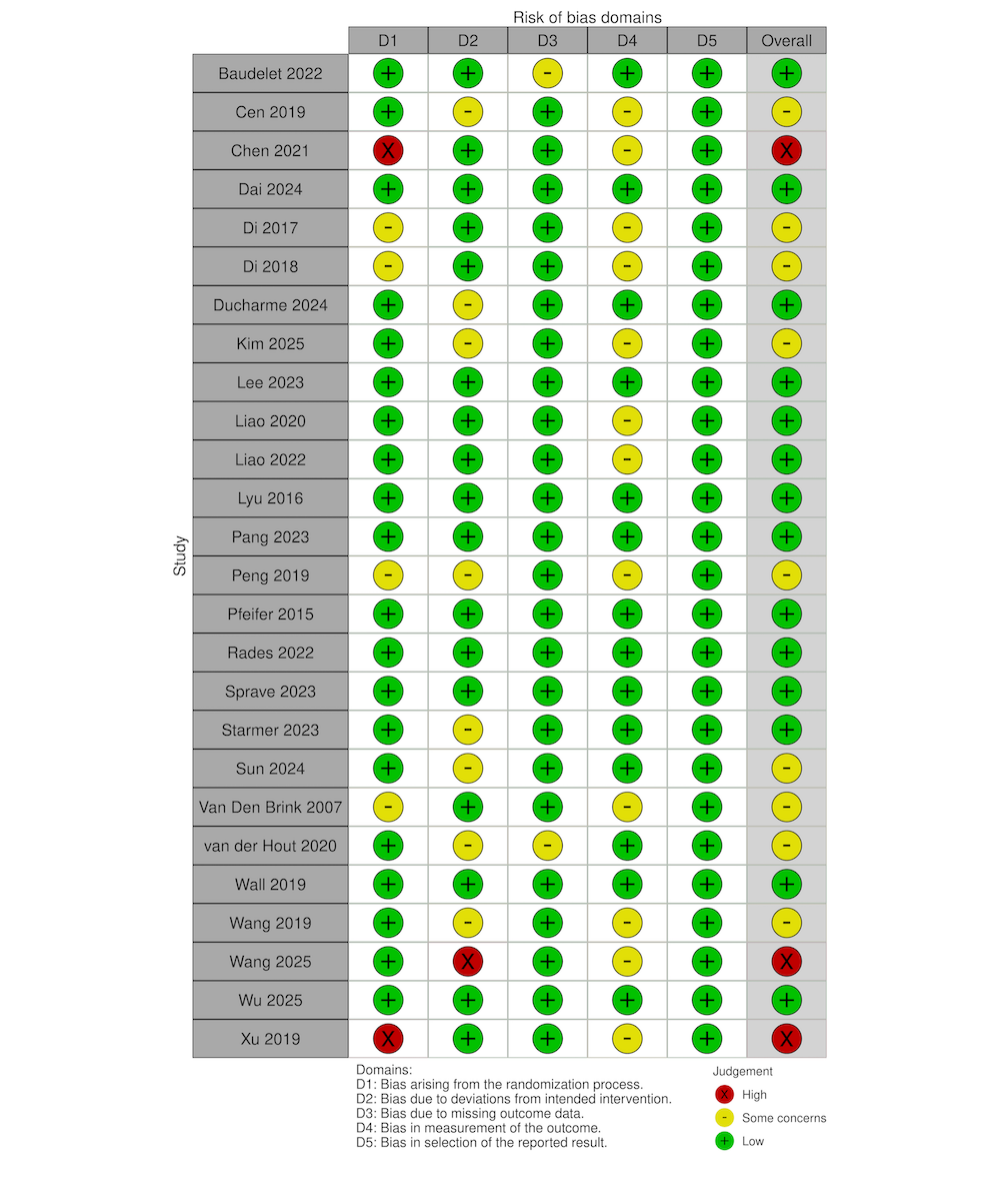
Risk of bias assessment [40-65].

Methodological quality was evaluated by two reviewers independently using the RoB 2.0 tool. The interrater consistency was high, with κ coefficients ranging from 0.82 to 1.00. Detailed results are presented in Figure 2. While all studies were RCTs, 8 did not specify the method of allocation concealment, resulting in “some concerns” in the randomization process. Due to the nature of mHealth interventions, blinding of participants and personnel was largely unfeasible; thus, most studies were rated as “some concerns” regarding deviations from intended interventions. Three studies were classified as “high risk” for attrition bias due to substantial missing data or high dropout rates. Regarding outcome measurement, only 6 studies reported the use of blinded assessors. Overall, 14 studies were rated as having a “low risk” of bias [40,43,46,48-52,54-57,61,64], 9 as “some concerns” [41,44,45,47,53,58-60,62],” and 3 as “high risk” [42,63,65].” Despite the risk of attrition in certain trials—often due to disease progression or treatment-related mortality—these studies were retained in the analysis to ensure a comprehensive synthesis of the current evidence. GRADE assessment of the 7 outcomes indicated that the overall quality of evidence rating ranged from low to very low (Multimedia Appendix 1). This was primarily due to the high risk of bias in most studies, substantial heterogeneity, and imprecision in certain results.

### Qualitative Synthesis of Findings

The included studies indicate that mHealth interventions for patients with HNC primarily focus on 4 domains: health education, self-monitoring, psychological support, and remote guidance. Most platforms adopted a multicomponent approach to facilitate self-management. For instance, Ducharme et al [46] used the “PTSD Coach” app, which integrated psychoeducation with coping tools for self-assessment and social support. Kim et al [47] developed “TOP note,” a theory-based application that combined goal setting with smartwatch-linked feedback to manage stress and sleep among thyroid cancer survivors. The degree of clinical interaction varied across platforms; for example, Baudelet et al [40] found that while app-based exercise programs provided clear digital instructions and reminders, the absence of real-time clinical feedback potentially attenuated therapeutic efficacy compared to traditional therapist-led modalities.

Regarding psychological outcomes, mHealth interventions showed significant potential. Ducharme et al [46] reported improved anxiety, depression, and QoL scores at the 3-month follow-up. Kim et al [47] observed significant reductions in perceived stress and improvements in both subjective and objective sleep quality. However, the evidence for physical rehabilitation was more heterogeneous. While mHealth tools were generally well-accepted, patient adherence remained a critical challenge, particularly during intensive treatment phases. Baudelet et al [40] noted that adherence to app-based swallowing exercises declined over time and was lower than that of face-to-face therapy.

Other studies emphasized different strengths of mHealth. Lyu et al [51] used WeChat for postdischarge follow-up and found it improved patient satisfaction, reduced anxiety, and supported continuity of care. Van Den Brink et al [59] used an early web-based system that helped detect serious complications early and gave patients a strong sense of security. Rades et al [55] tested a simple reminder app for skin and mouth care during radiotherapy; while it did not reach statistical significance, it showed a trend toward fewer severe side effects. Additionally, Wall et al [61] showed that the digital swallowing therapy program “SwallowIT” was clinically equivalent to traditional in-person therapy, suggesting it serves as a viable and practical alternative for HNC rehabilitation.

### Meta-Analysis Results

#### Quality of Life

**Figure 3.**
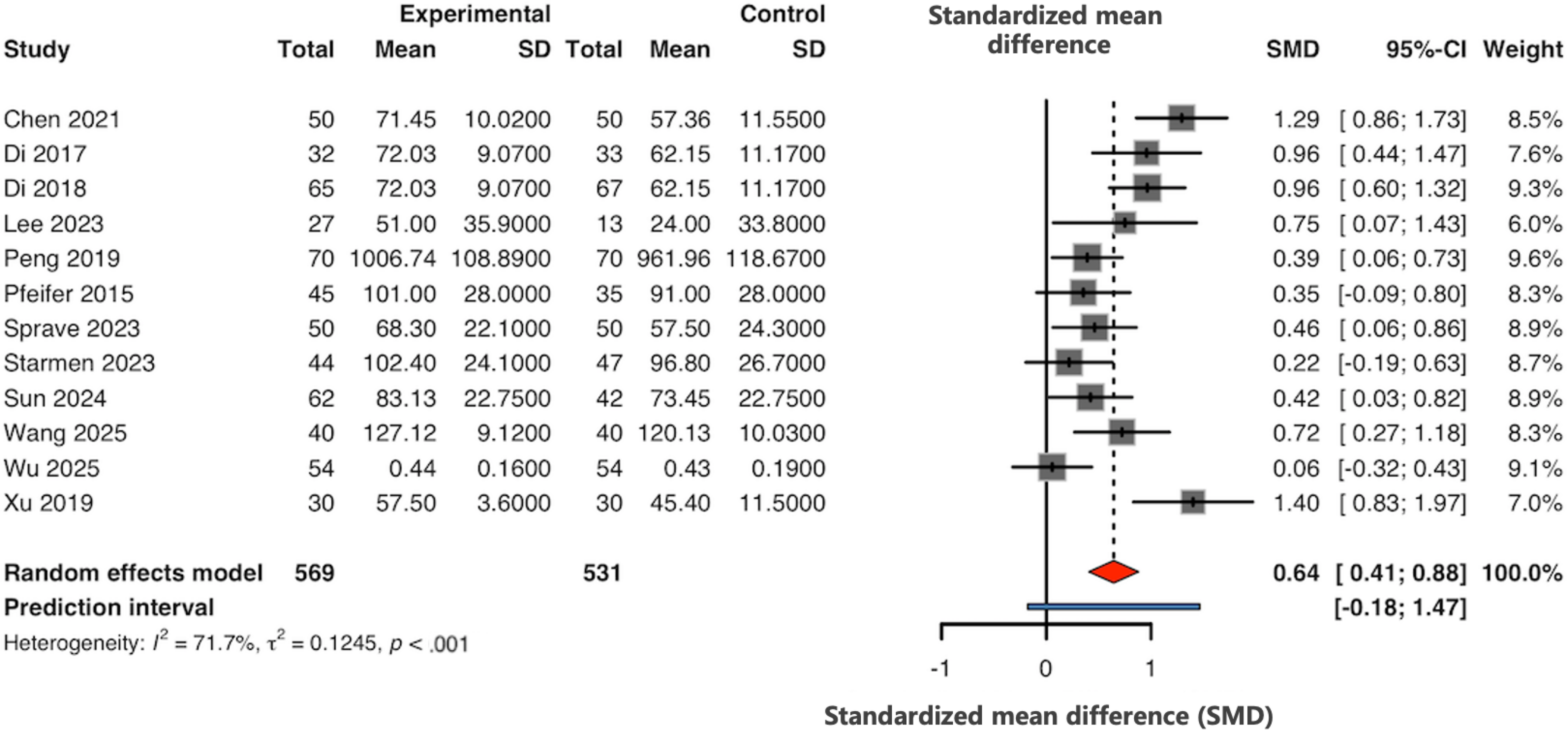
Meta-analysis on quality of life [42,44,45,48,53,54,56-58,63-65].

A total of 12 (46%) studies [42,44,45,48,53,54,56-58,63-65] (46%) provided data on QOL. The pooled results indicated that mHealth interventions had a significant positive impact on the QOL of patients with HNC compared to routine care (SMD 0.64, 95% CI 0.41-0.88; *I*^2^=72%) (Figure 3). However, the 95% PI ranged from −0.18 to 1.47, suggesting that the impact of mHealth on QOL may vary across different clinical settings.

#### Anxiety and Depression

**Figure 4.**
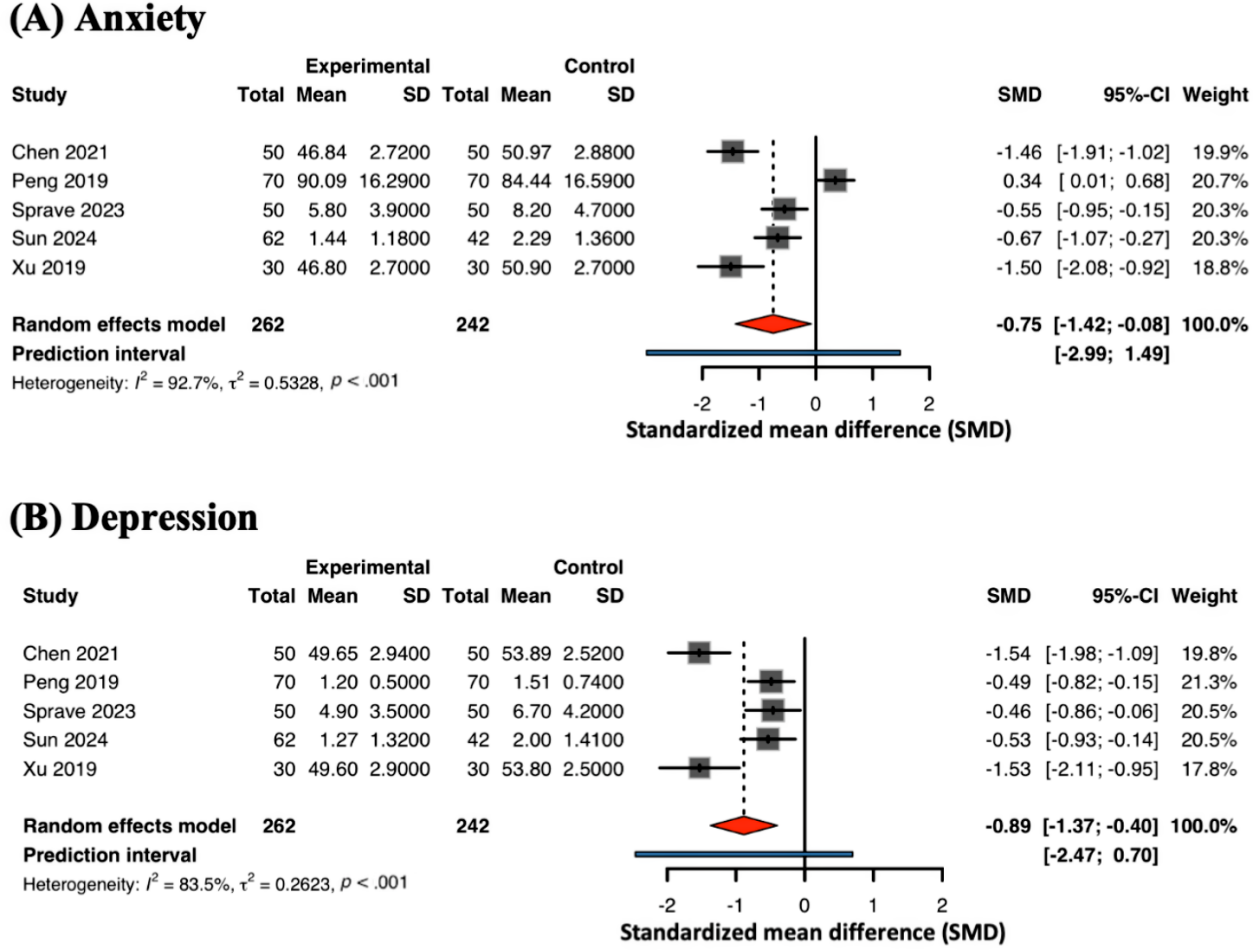
Meta-analysis on (A) anxiety and (B) depression [42,53,56,58,65].

Five studies [42,53,56,58,65] (19%) evaluated the effect of mHealth on psychological outcomes (Figure 4). The meta-analysis demonstrated that mHealth interventions significantly alleviated anxiety symptoms (SMD −0.75, 95% CI −1.42 to −0.08; *I*^2^=93%). Due to extreme heterogeneity, the 95% PI was wide and encompassed the null value (−2.99 to 1.49), indicating that the intervention’s effect on anxiety remains highly variable across different populations. Similarly, 5 studies reported depression outcomes, showing a significant reduction in depressive symptoms (SMD −0.89, 95% CI −1.37 to −0.40; *I*^2^=84%), with a 95% PI of −2.47 to 0.70. These findings suggest that digital platforms are effective on average, though the wide PIs necessitate caution in generalizing these results.

#### Fatigue and Pain

**Figure 5.**
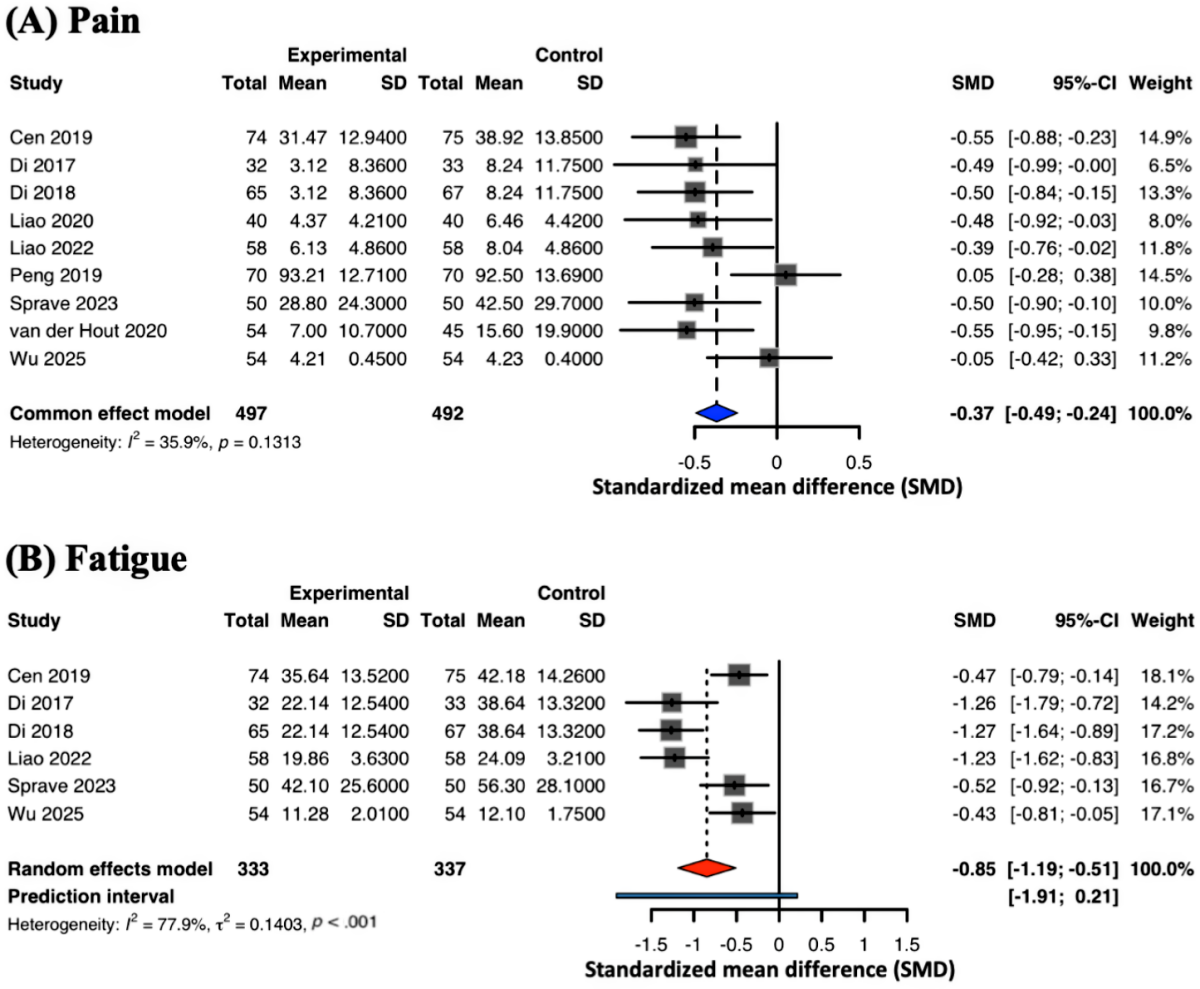
Meta-analysis on (A) pain and (B) fatigue [41,44,45,49,50,53,56,60,64].

Fatigue was evaluated in 6 studies [41,44,45,49,56,64] (23%), with the pooled estimate showing a significant beneficial effect of mHealth interventions (SMD −0.85, 95% CI −1.19 to −0.51; *I*^2^=78%) (Figure 5). The 95% PI was −1.91 to 0.21, reflecting considerable variation in expected fatigue reduction.

Nine studies [41,44,45,49,50,53,56,60,64] (35%) assessed pain intensity, and the meta-analysis revealed a small but statistically significant reduction in pain scores (SMD −0.37, 95% CI −0.49 to −0.24; *I*^2^=36%) (Figure 5). The lower heterogeneity in this outcome supports a more consistent effect across studies.

#### Swallowing Function

**Figure 6.**
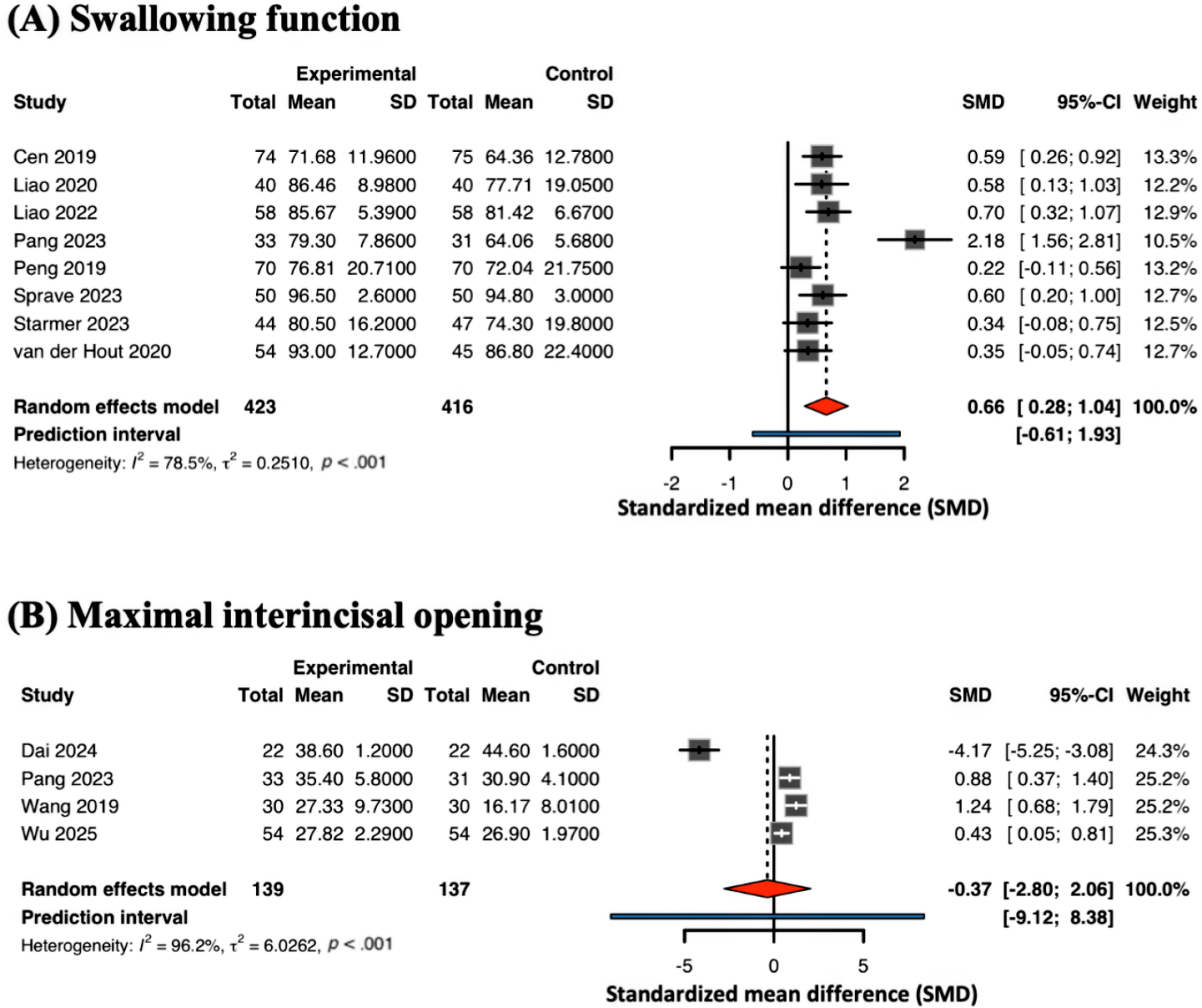
Meta-analysis on (A) swallowing function and (B) maximal interincisal opening [41,43,49,50,52,53,56,57,60,62,64].

Eight studies [41,49,50,52,53,56,57,60] (31%) reported on swallowing function. The improvement in swallowing function showed borderline significance (SMD 0.66, 95% CI 0.28-1.04; *I*^2^=91%) (Figure 6), suggesting that the current evidence is fragile and should be interpreted with caution. The 95% PI ranged from −0.61 to 1.93, which indicates that the “average” effect may not be clinically representative for all future patients, and the intervention’s efficacy remains variable.

#### Maximal Interincisal Opening

Four studies [43,52,62,64] (15%) examined MIO as an outcome. The meta-analysis found no statistically significant difference between mHealth and control groups (SMD −0.37, 95% CI −2.80 to 2.06; *P*=.68; *I*^2^=96%) (Figure 6). The wide confidence interval and very high heterogeneity indicate inconsistency across studies, likely due to differences in intervention intensity, follow-up duration, and baseline function. Pooled effect sizes and 95% CIs for all examined outcomes are summarized in Multimedia Appendix 1.

### Subgroup Analysis

As shown in Table 2, subgroup analyses were conducted across outcomes, including anxiety, depression, fatigue, pain, swallow function, and QOL. We stratified the data by assessment scale, intervention format, and intervention duration. Additionally, we examined the impact of specific components, including telemedical support, self-monitoring, and home practice support. Detailed results for each outcome are presented in Multimedia Appendix 1.

Home practice support significantly reduced symptoms of anxiety (SMD −1.48, 95% CI −1.83 to −1.12; *P*<.001; *I*^2^=0%) and depression (SMD −1.53, 95% CI −1.88 to −1.18; *P*<.001; *I*^2^=0%), while simultaneously enhancing QoL (SMD 0.72, 95% CI 0.19-1.25; *P*=.008; *I*^2^=86%). Self-monitoring was strongly associated with better swallowing function (SMD 0.50, 95% CI 0.31-0.70; *P*<.001; *I*^2^=0%). The therapeutic impact was found to be moderated by intervention duration. Short-term interventions (<3 months) showed significant effects on both QoL (SMD 0.68, 95% CI 0.38-0.98; *P*<.001; *I*^2^=70%) and swallowing function (SMD 0.59, 95% CI 0.34-0.85; *P*<.001; *I*^2^=0%). Long-term interventions (≥3 months) remained effective for QoL (SMD 0.74, 95% CI 0.44-1.03; *P*<.001; *I*^2^=52%). In contrast, while the pooled effect size for swallowing function appeared positive, it failed to reach statistical significance (SMD 0.42, 95% CI –0.18 to 1.02; *P*=.17; *I*^2^=93%). However, interventions without telemedical support showed stronger effects across all outcomes compared to those with such support (*P*>.05).

**Table 2.** Subgroup analyses of mobile health intervention efficacy.

Subgroup variable	Studies, n	SMD^a^ (95% CI)	*P* value	*I*^2^ (%)
Anxiety				
Home practice support	2	–1.48 (–1.83 to –1.12)	<.001	0
Self-monitoring	2	–1.00 (–1.93 to –0.07)	.03	86
Telemedical support	3	–0.55 (–1.53 to 0.44)	<.001	85
Without telemedical support	2	–1.06 (–1.84 to –0.29)	.03	85
Depression				
Home practice support	2	–1.53 (–1.88 to –1.18)	<.001	0
Telemedical support	2	–0.48 (–0.77 to –0.19)	.003	83
Without telemedical support	3	–1.18 (–1.48 to –0.89)	<.001	83
Swallowing function				
Self-monitoring	4	0.50 (0.31 to 0.70)	<.001	0
Duration: short-term (≤3 months)	2	0.59 (0.34 to 0.85)	<.001	0
Duration: long-term (>3 months)	7	0.42 (–0.18 to 1.02)	.17	93
Scale: EORTC^b^-based	5	0.57 (0.40 to 0.74)	<.001	0
Quality of life				
Home practice support	5	0.72 (0.19 to 1.25)	<.001	86
Duration: short-term (≤3 months)	7	0.68 (0.38 to 0.98)	<.001	70
Duration: long-term (>3 months)	4	0.74 (0.44 to 1.03)	<.001	52
Pain				
Self-monitoring	4	–0.48 (–0.66 to –0.31)	<.001	65
Scale: EORTC-based	6	–0.50 (–0.64 to –0.35)	<.001	0
With telemedical support	4	–0.34 (–0.52 to –0.16)	<.001	62
Without telemedical support	4	–0.39 (–0.56 to –0.22)	<.001	10

a SMD: Standardized mean difference.

b EORTC: European Organisation for Research and Treatment of Cancer.

### Sensitivity Analysis

The sensitivity analyses for the meta-analysis results of anxiety, depression, fatigue, QoL, and pain showed that the pooled estimates remained robust when any single study was excluded. However, swallowing function reached only borderline significance in the random-effects model and was not robust in sensitivity analysis. The results of MIO revealed that after excluding the study by Dai et al [43], the overall pooled result changed from nonstatistically significant (SMD −0.37, 95% CI −2.80 to 2.06; *P*=.68; *I*^2^=96%) to statistically significant (SMD 0.81, 95% CI 0.34-1.28; *P*<.001; *I*^2^=66%). Detailed sensitivity analysis results are presented in Multimedia Appendix 1.

### Publication Bias

Publication bias was assessed using funnel plots and Egger tests including QoL, pain, and swallowing function. For other indicators, the number of included studies was insufficient to perform these tests. Visual inspection of the funnel plots showed general symmetry, and Egger tests revealed no evidence of significant publication bias for QoL (*P*=.59), pain (*P*=.78), or swallowing function (*P*=.91) (Multimedia Appendix 1).

## Discussion

### Principal Findings

HNC survivors often suffer from persistent functional deficits and psychological distress. Compared with other cancers, they require specialized and continuous rehabilitation after discharge. With the rapid advancement of digital health technologies, mHealth has been an increasingly used approach to support self-management and improve health outcomes in cancer care. This systematic review and meta-analysis provides evidence-based data regarding the efficacy of mHealth in enhancing QoL and alleviating symptoms of anxiety, depression, fatigue, pain, and swallowing function among patients with HNC. Furthermore, we analyzed the influence of various intervention formats and durations on these outcomes, providing evidence for the development of optimized digital health protocols.

Our findings indicate that mHealth interventions significantly enhance QoL and alleviate psychological distress, such as anxiety and depression, aligning with results from previous systematic reviews [17,18,24]. These improvements are primarily attributed to real-time psychological support, peer interaction, and continuous symptom tracking, which help reduce uncertainty about the illness and lower the psychological burden during rehabilitation. Evidence suggests that the mitigation of anxiety and depression is concurrently associated with improved QoL [66]. Despite variations in intervention formats, durations, and target populations, sensitivity analyses confirmed the robustness of these positive psychosocial outcomes. Sun et al [58] used an intervention based on the multitheory model and found significant improvements in anxiety, fear of cancer recurrence, and overall QoL, but no meaningful changes in physical function or somatic symptoms. These results indicate that the primary benefit of such interventions lies in addressing the psychological and emotional challenges faced by cancer survivors. Furthermore, subgroup analyses revealed that “home practice support” is associated with robust improvements in anxiety, depression, and QoL. This indicates that integrating psychological support with specific rehabilitation tasks can enhance self-efficacy and behavioral adherence [58]. The sustained improvement in QoL observed in long-term interventions (≥3 months) further highlights the importance of mHealth in maintaining long-term well-being.

Studies report that pain and fatigue are the most prominent physical symptoms among patients with HNC, particularly in postoperative populations. Our findings indicate that mHealth interventions lead to moderate improvements in both symptoms; specifically, the alleviation of fatigue was relatively pronounced, whereas the reduction in pain was more limited. These findings align with previous systematic reviews [67]. For instance, Hernandez Silva et al [67] reported that self-management-based mHealth interventions effectively improved fatigue in cancer survivors but failed to reach statistical significance for pain symptoms. Among the studies included in this analysis, interventions using self-management applications or WeChat-based platforms showed superior effects in mitigating fatigue and pain [49,56,64]. This efficacy may be attributed to the integration of personalized reminders, symptom tracking, and real-time feedback, which collectively enhance patient self-management capabilities and treatment adherence.

Dysphagia and trismus are common complications following HNC treatment [68]. These impairments can lead to malnutrition, dehydration, weight loss, chronic aspiration, and aspiration pneumonia, significantly affecting patients’ QoL and long-term prognosis. Although this review did not observe an overall significant improvement in MIO through mHealth, the results indicated that self-monitoring significantly enhanced swallowing outcomes. Encouraging patients to actively record and monitor their swallowing status may enhance rehabilitation awareness and behavioral adherence [22]. In addition, the effects on swallowing function were more pronounced in short-term interventions (<3 months), highlighting the importance of early intervention, which aligns with the findings of a recent systematic review [24]. Current evidence on MIO is highly inconsistent and sensitive to individual study inclusion, warranting cautious interpretation. Future research should prioritize large-scale, high-quality trials focused on dysphagia and trismus. Moreover, the long-term benefits of mHealth on these functional outcomes require further validation through rigorous longitudinal studies.

Interestingly, the subgroup analysis showed that interventions without telemedical support appeared to be more effective. This finding is counterintuitive as it challenges the assumption that “human-in-the-loop” interventions are inherently more effective than fully automated systems. For example, some “telemedical” support was limited to low-intensity and noninteractive notifications, which might increase a patient’s cognitive load without providing meaningful clinical feedback. In contrast, simple tools like WeChat may be more successful because they are easier to use in daily life. However, a note of caution is necessary when interpreting this finding. This result may be a statistical artifact specific to the limited number of studies in these subgroups rather than a general rule that “less is more” in mHealth. The better results seen in automated systems likely reflect the high quality of their content and fewer technical difficulties, rather than a disadvantage of human interaction. Future research should focus on identifying which types of human involvement truly contribute to clinical value in HNC rehabilitation.

The extreme heterogeneity observed in outcomes such as anxiety and swallowing function has critical clinical implications. While the pooled SMDs suggest an overall benefit, the 95% PIs for these outcomes encompass the null value. This indicates that the “average” effect may not be clinically representative of every setting, and the results should be interpreted with extreme caution. The observed variance primarily stems from diverse intervention designs (eg, WeChat vs specialized apps), patient population variability (eg, tumor sites and treatment modalities), and inconsistent assessment tools. These findings underscore the need for standardized intervention protocols to better validate the long-term clinical and economic value of mHealth in HNC rehabilitation.

### Implications for Clinical Practice

Our findings provide clear guidance for mHealth apps in HNC rehabilitation. Health care professionals should use mHealth to enhance symptom management and QoL, particularly during the first 3 months posttreatment. Effective mHealth tool design should prioritize “home practice support” and “self-monitoring” functions. Using concise mini-programs or lightweight apps is generally more effective and less burdensome than complex teleconsultation systems. Furthermore, interventions should be based on established behavior change theories and tailored to specific tumor sites and treatment modalities. An ideal care model involves a “hospital-community-home” integrated system, where mHealth platforms facilitate daily tracking and structured training, supported by periodic multidisciplinary feedback. Beyond automated education, platforms should incorporate features for private inquiries and personalized responses. Finally, addressing barriers for older patients and those with low digital literacy through age-appropriate design is essential to ensure equity. Future research requires high-quality, large-scale RCTs to validate functional outcomes, along with systematic evaluations of long-term efficacy and health economic benefits across diverse HNC subtypes and cultural backgrounds.

### Strengths and Limitations

This study has several strengths. First, our comprehensive search across multiple databases ensured extensive literature coverage. Second, we exclusively included RCTs, and most of the selected trials had a low risk of bias. In addition, we evaluated both psychosocial and physiological outcomes by detailed subgroup analyses.

Despite these strengths, several limitations must be acknowledged. First, the generalizability of our findings is limited by geographic and platform concentration. Nearly half of the included studies rely on 2 specific platforms: WeChat in China and OncoKompas in the Netherlands. These tools depend on localized digital infrastructures that may not be available elsewhere. Second, most evidence comes from high- or middle-income settings with high smartphone penetration. Consequently, our results may not apply to patients in low-resource settings, rural areas, or low- and middle-income countries. Finally, substantial heterogeneity was observed, and the overall quality of evidence for several outcomes remains low to very low.

### Conclusions

mHealth interventions are a promising adjunct for improving QoL and alleviating psychological distress, fatigue, and pain in patients with HNC. Home practice support and self-monitoring are pivotal components for driving efficacy. Future research should prioritize high-quality, large-scale RCTs to further validate the long-term effectiveness and standardized implementation of these digital interventions.

## Supplementary material

10.2196/78109 Multimedia Appendix 1 Search strategies, GRADE (Grading of Recommendations, Assessment, Development, and Evaluation) evidence profiles, intervention characteristics, and supplementary statistical analyses, including subgroup, sensitivity, and publication bias analyses.

10.2196/78109 Checklist 1 PRISMA checklist.

## Abbreviations

EORTC QLQ-C30: European Organisation for Research and Treatment of Cancer Quality of Life Questionnaire Core 30
EORTC QLQ-H&N35: European Organisation for Research and Treatment of Cancer Quality of Life Questionnaire Head and Neck 35
GRADE: Grading of Recommendations, Assessment, Development, and Evaluation
HNC: head and neck cancer
mHealth: mobile health
MIO: maximal interincisal opening
PI: prediction interval
PICOS: participants, intervention, comparison, outcome, and study design
PRISMA: Preferred Reporting Items for Systematic Reviews and Meta-Analyses
QoL: quality of life
RCT: randomized controlled trial
RoB 2.0: Cochrane Risk of Bias 2.0
SMD: standardized mean difference
