# Supplementary material for: Evaluating Effectiveness of mHealth Interventions for Rehabilitation in Patients With Head and Neck Cancer: Systematic Review and Meta-Analysis of Randomized Controlled Trials
Source: JMIR Mhealth Uhealth. 2026 Apr 30;14:e78109. doi: 10.2196/78109 (PMC13131825; doi:10.2196/78109)
Supplement: Multimedia Appendix 1 [file mhealth-v14-e78109-s001.docx]

# The Syntax of the Search Strategy

Searched from inception to December 1, 2025

| Database | Search Strategies | Results |
| --- | --- | --- |
| PubMed | (("head and neck cancer"[Title/Abstract] OR "oropharyngeal cancer"[Title/Abstract] OR "laryngeal cancer"[Title/Abstract] OR "salivary gland cancer"[Title/Abstract] OR "hypopharyngeal cancer"[Title/Abstract] OR "nasopharyngeal cancer"[Title/Abstract] OR "oral cancer"[Title/Abstract] OR "paranasal sinus cancer"[Title/Abstract] OR "nasal cavity cancer"[Title/Abstract] OR "head and neck carcinoma"[Title/Abstract] OR "head and neck tumor"[Title/Abstract] OR "head and neck malignancy"[Title/Abstract] OR "HNC"[Title/Abstract]) AND ("App"[Title/Abstract] OR "Application"[Title/Abstract] OR "Cell Phone"[Title/Abstract] OR "Computer"[Title/Abstract] OR "Digital health"[Title/Abstract] OR "Digital health application"[Title/Abstract] OR "Digital technolog*"[Title/Abstract] OR "e-health"[Title/Abstract] OR "eHealth"[Title/Abstract] OR "Email"[Title/Abstract] OR "Health app"[Title/Abstract] OR "Internet"[Title/Abstract] OR "Internet-Based Intervention"[Title/Abstract] OR "m-health"[Title/Abstract] OR "mHealth"[Title/Abstract] OR "mHealth app"[Title/Abstract] OR "Mobile"[Title/Abstract] OR "Mobile Application"[Title/Abstract] OR "Mobile device"[Title/Abstract] OR "Mobile health"[Title/Abstract] OR "Mobile health app"[Title/Abstract] OR "Mobile health application"[Title/Abstract] OR "Mobile health technology"[Title/Abstract] OR "Mobile intervention"[Title/Abstract] OR "Mobile phone"[Title/Abstract] OR "Mobile-based intervention"[Title/Abstract] OR "Online"[Title/Abstract] OR "Phone"[Title/Abstract] OR "Remote health monitoring"[Title/Abstract] OR "Smartphone"[Title/Abstract] OR "Smartphone health app"[Title/Abstract] OR "Technology"[Title/Abstract] OR "Telemedicine"[Title/Abstract] OR "Telemedicine app"[Title/Abstract] OR "Telephone"[Title/Abstract] OR "Telerehabilitation"[Title/Abstract] OR "Text messaging"[Title/Abstract] OR "Video"[Title/Abstract] OR "Web"[Title/Abstract] OR "Website"[Title/Abstract]) AND (2000:2026[pdat])) AND (("RCT"[Title/Abstract] OR "randomized controlled trial"[Title/Abstract] OR "randomized clinical trial"[Title/Abstract] OR "randomized trial"[Title/Abstract] OR "randomised controlled trial"[Title/Abstract] OR "randomised trial"[Title/Abstract] OR "randomization"[Title/Abstract] OR "randomisation"[Title/Abstract] OR "randomly allocated"[Title/Abstract])) | 170 |
| Embase | ('head and neck cancer'/exp OR 'oropharyngeal cancer'/exp OR 'laryngeal cancer'/exp OR 'salivary gland cancer'/exp OR 'hypopharyngeal cancer'/exp OR 'nasopharyngeal cancer'/exp OR 'oral cancer'/exp OR 'paranasal sinus cancer'/exp OR 'nasal cavity cancer'/exp OR 'head and neck cancer':ti,ab OR 'oropharyngeal cancer':ti,ab OR 'laryngeal cancer':ti,ab OR 'salivary gland cancer':ti,ab OR 'hypopharyngeal cancer':ti,ab OR 'nasopharyngeal cancer':ti,ab OR 'oral cancer':ti,ab OR 'paranasal sinus cancer':ti,ab OR 'nasal cavity cancer':ti,ab OR 'head and neck carcinoma':ti,ab OR 'head and neck tumor':ti,ab OR 'head and neck malignancy':ti,ab OR 'HNC':ti,ab) AND ('telemedicine'/exp OR 'mobile application'/exp OR 'internet'/exp OR 'ehealth'/exp OR 'remote health monitoring'/exp OR 'health information system'/exp OR 'text messaging'/exp OR 'telephone'/exp OR 'telerehabilitation'/exp OR 'App':ti,ab OR 'Application':ti,ab OR 'Cell Phone':ti,ab OR 'Computer':ti,ab OR 'Digital health':ti,ab OR 'Digital health application':ti,ab OR 'Digital technolog*':ti,ab OR 'e-health':ti,ab OR 'eHealth':ti,ab OR 'Email':ti,ab OR 'Health app':ti,ab OR 'Internet':ti,ab OR 'Internet-Based Intervention':ti,ab OR 'm-health':ti,ab OR 'mHealth':ti,ab OR 'mHealth app':ti,ab OR 'Mobile':ti,ab OR 'Mobile Application':ti,ab OR 'Mobile device':ti,ab OR 'Mobile health':ti,ab OR 'Mobile health app':ti,ab OR 'Mobile health application':ti,ab OR 'Mobile health technology':ti,ab OR 'Mobile intervention':ti,ab OR 'Mobile phone':ti,ab OR 'Mobile-based intervention':ti,ab OR 'Online':ti,ab OR 'Phone':ti,ab OR 'Remote health monitoring':ti,ab OR 'Smartphone':ti,ab OR 'Smartphone health app':ti,ab OR 'Technology':ti,ab OR 'Telemedicine':ti,ab OR 'Telemedicine app':ti,ab OR 'Telephone':ti,ab OR 'Telerehabilitation':ti,ab OR 'Text messaging':ti,ab OR 'Video':ti,ab OR 'Web':ti,ab OR 'Website':ti,ab) AND ('randomized controlled trial'/exp OR 'controlled clinical trial'/exp OR 'RCT':ti,ab OR 'randomized clinical trial':ti,ab OR 'randomized controlled trial':ti,ab OR 'randomized trial':ti,ab OR 'randomised controlled trial':ti,ab OR 'randomised trial':ti,ab OR 'randomization':ti,ab OR 'randomisation':ti,ab) | 1791 |
| Cochrane Central Register of Controlled Trials | ("App" OR "Application" OR "Cell Phone" OR "Computer" OR "Digital health" OR "Digital health application" OR "Digital technolog*" OR "e-health" OR "eHealth" OR "Email" OR "Health app" OR "Internet" OR "Internet-Based Intervention" OR "m-health" OR "mHealth" OR "mHealth app" OR "Mobile" OR "Mobile Application" OR "Mobile device" OR "Mobile health" OR "Mobile health app" OR "Mobile health application" OR "Mobile health technology" OR "Mobile intervention" OR "Mobile phone" OR "Mobile-based intervention" OR "Online" OR "Phone" OR "Remote health monitoring" OR "Smartphone" OR "Smartphone health app" OR "Technology" OR "Telemedicine" OR "Telemedicine app" OR "Telephone" OR "Telerehabilitation" OR "Text messaging" OR "Video" OR "Web" OR "Website") in Title Abstract Keyword AND ("head and neck cancer" OR "oropharyngeal cancer" OR "laryngeal cancer" OR "salivary gland cancer" OR "hypopharyngeal cancer" OR "nasopharyngeal cancer" OR "oral cancer" OR "paranasal sinus cancer" OR "nasal cavity cancer" OR "head and neck carcinoma" OR "head and neck tumor" OR "head and neck malignancy" OR "HNC") in Title Abstract Keyword AND ("RCT" OR "randomized clinical trial" OR "randomized controlled trial" OR "randomized trial" OR "randomised controlled trial" OR "randomised trial") in Title Abstract Keyword | 699 |
| Web of Science | (TS=("head and neck cancer" OR "oropharyngeal cancer" OR "laryngeal cancer" OR "salivary gland cancer" OR "hypopharyngeal cancer" OR "nasopharyngeal cancer" OR "oral cavity cancer" OR "paranasal sinus cancer" OR "nasal cavity cancer" OR "head and neck carcinoma" OR "head and neck tumor" OR "head and neck malignancy" OR "HNC") AND (TS=("mobile health application" OR "mHealth" OR "mobile health app" OR "telemedicine app" OR "health app" OR "digital health application" OR "smartphone health app" OR "remote health monitoring" OR "eHealth" OR "mobile intervention" OR "mobile-based intervention" OR "mobile health technology") | 314 |
| Google scholar | ("App" OR "Application" OR "Cell Phone" OR "Computer" OR "Digital health" OR "Digital health application" OR "Digital technolog*" OR "e-health" OR "eHealth" OR "Email" OR "Health app" OR "Internet" OR "Internet-Based Intervention" OR "m-health" OR "mHealth" OR "mHealth app" OR "Mobile" OR "Mobile Application" OR "Mobile device" OR "Mobile health" OR "Mobile health app" OR "Mobile health application" OR "Mobile health technology" OR "Mobile intervention" OR "Mobile phone" OR "Mobile-based intervention" OR "Online" OR "Phone" OR "Remote health monitoring" OR "Smartphone" OR "Smartphone health app" OR "Technology" OR "Telemedicine" OR "Telemedicine app" OR "Telephone" OR "Telerehabilitation" OR "Text messaging" OR "Video" OR "Web" OR "Website") AND ("head and neck cancer" OR "oropharyngeal cancer" OR "laryngeal cancer" OR "salivary gland cancer" OR "hypopharyngeal cancer" OR "nasopharyngeal cancer" OR "oral cancer" OR "paranasal sinus cancer" OR "nasal cavity cancer" OR "head and neck carcinoma" OR "head and neck tumor" OR "head and neck malignancy" OR "HNC") AND ("RCT" OR "randomized clinical trial" OR "randomized controlled trial" OR "randomized trial" OR "randomised controlled trial" OR "randomised trial") | 790 |
| CINAHL | ((MH "Head and Neck Neoplasms") OR (MH "Otorhinolaryngologic Neoplasms") OR ("head and neck cancer" OR "oropharyngeal cancer" OR "laryngeal cancer" OR "salivary gland cancer" OR "hypopharyngeal cancer" OR "nasopharyngeal cancer" OR "oral cancer" OR "paranasal sinus cancer" OR "nasal cavity cancer" OR "head and neck carcinoma" OR "head and neck tumor" OR "head and neck malignancy" OR "HNC").ti,ab.) AND ((MH "Mobile Applications") OR (MH "Telemedicine") OR (MH "Internet-Based Interventions") OR (MH "Text Messaging") OR ("App" OR "Application" OR "Cell Phone" OR "Computer" OR "Digital health" OR "Digital health application" OR "Digital technolog*" OR "e-health" OR "eHealth" OR "Email" OR "Health app" OR "Internet" OR "Internet-Based Intervention" OR "m-health" OR "mHealth" OR "mHealth app" OR "Mobile" OR "Mobile Application" OR "Mobile device" OR "Mobile health" OR "Mobile health app" OR "Mobile health application" OR "Mobile health technology" OR "Mobile intervention" OR "Mobile phone" OR "Mobile-based intervention" OR "Online" OR "Phone" OR "Remote health monitoring" OR "Smartphone" OR "Smartphone health app" OR "Technology" OR "Telemedicine app" OR "Telephone" OR "Telerehabilitation" OR "Text messaging" OR "Video" OR "Web" OR "Website").ti,ab.) AND ((MH "Randomized Controlled Trials") OR ("RCT" OR "randomized clinical trial" OR "randomized controlled trial" OR "randomized trial" OR "randomised controlled trial" OR "randomised trial").ti,ab.) | 297 |
| PsycINFO | ((("head and neck cancer" or "oropharyngeal cancer" or "laryngeal cancer" or "salivary gland cancer" or "hypopharyngeal cancer" or "nasopharyngeal cancer" or "oral cancer" or "paranasal sinus cancer" or "nasal cavity cancer" or "head and neck carcinoma" or "head and neck tumor" or "head and neck malignancy" or "HNC").ti,ab. or "Head and Neck Neoplasms"/ or "Otorhinolaryngologic Neoplasms"/)) AND (("App" or "Application" or "Cell Phone" or "Computer" or "Digital health" or "Digital health application" or "Digital technolog*" or "e-health" or "eHealth" or "Email" or "Health app" or "Internet" or "Internet-Based Intervention" or "m-health" or "mHealth" or "mHealth app" or "Mobile" or "Mobile Application" or "Mobile device" or "Mobile health" or "Mobile health app" or "Mobile health application" or "Mobile health technology" or "Mobile intervention" or "Mobile phone" or "Mobile-based intervention" or "Online" or "Phone" or "Remote health monitoring" or "Smartphone" or "Smartphone health app" or "Technology" or "Telemedicine" or "Telemedicine app" or "Telephone" or "Telerehabilitation" or "Text messaging" or "Video" or "Web" or "Website").ti,ab. or "Mobile Health"/ or "Telemedicine"/ or "Internet-Based Intervention"/)) AND (("RCT" or "randomized clinical trial" or "randomized controlled trial" or "randomized trial" or "randomised controlled trial" or "randomised trial").ti,ab. or "Randomized Controlled Trial"/ or "Controlled Clinical Trial"/)) | 145 |
| Scopus | (TITLE-ABS-KEY("head and neck cancer" OR "oropharyngeal cancer" OR "laryngeal cancer" OR "salivary gland cancer" OR "hypopharyngeal cancer" OR "nasopharyngeal cancer" OR "oral cancer" OR "paranasal sinus cancer" OR "nasal cavity cancer" OR "head and neck carcinoma" OR "head and neck tumor" OR "head and neck malignancy" OR "HNC")) AND (TITLE-ABS-KEY("App" OR "Application" OR "Cell Phone" OR "Computer" OR "Digital health" OR "Digital health application" OR "Digital technolog*" OR "e-health" OR "eHealth" OR "Email" OR "Health app" OR "Internet" OR "Internet-Based Intervention" OR "m-health" OR "mHealth" OR "mHealth app" OR "Mobile" OR "Mobile Application" OR "Mobile device" OR "Mobile health" OR "Mobile health app" OR "Mobile health application" OR "Mobile health technology" OR "Mobile intervention" OR "Mobile phone" OR "Mobile-based intervention" OR "Online" OR "Phone" OR "Remote health monitoring" OR "Smartphone" OR "Smartphone health app" OR "Technology" OR "Telemedicine" OR "Telemedicine app" OR "Telephone" OR "Telerehabilitation" OR "Text messaging" OR "Video" OR "Web" OR "Website")) AND (TITLE-ABS-KEY("RCT" OR "randomized clinical trial" OR "randomized controlled trial" OR "randomized trial" OR "randomised controlled trial" OR "randomised trial")) | 134 |
| CNKI | (头颈癌 + 头颈肿瘤 + 头颈部癌 + 头颈部恶性肿瘤 + 口腔癌 + 喉癌 + 鼻咽癌) AND (移动医疗 + 远程医疗 + 手机 + 应用) AND (随机对照试验 + RCT) | 141 |
| WanFang | ( 摘要: 头颈癌 OR头颈肿瘤 OR头颈部癌 OR 头颈部恶性肿瘤 OR 口腔癌 OR 喉癌 OR 鼻咽癌) and ( 摘要: 移动医疗 OR远程医疗 OR 手机 OR 应用) | 22 |
| VIP | ( (篇名:头颈癌 OR 摘要:头颈癌 OR 关键词:头颈癌 OR 主题:头颈癌) OR (篇名:头颈肿瘤 OR 摘要:头颈肿瘤 OR 关键词:头颈肿瘤 OR 主题:头颈肿瘤) OR (篇名:口腔癌 OR 摘要:口腔癌 OR 关键词:口腔癌 OR 主题:口腔癌) OR (篇名:鼻咽癌 OR 摘要:鼻咽癌 OR 关键词:鼻咽癌 OR 主题:鼻咽癌) ) 并且 ( (篇名:移动医疗 OR 摘要:移动医疗 OR 关键词:移动医疗 OR 主题:移动医疗) OR (篇名:远程医疗 OR 摘要:远程医疗 OR 关键词:远程医疗 OR 主题:远程医疗) OR (篇名:手机应用 OR 摘要:手机应用 OR 关键词:手机应用 OR 主题:手机应用) OR (篇名:App OR 摘要:App OR 关键词:App) OR (篇名:eHealth OR 摘要:eHealth OR 关键词:eHealth) ) | 56 |
| CBM | (摘要='头颈癌' OR 摘要='头颈肿瘤' OR 摘要='头颈部癌' OR 摘要='头颈部恶性肿瘤' OR 摘要='口腔癌' OR 摘要='喉癌' OR 摘要='鼻咽癌') 并且 (摘要='移动医疗' OR 摘要='远程医疗' OR 摘要='手机' OR 摘要='应用') | 39 |

# Grading of Recommendations, Assessment, Development, and Evaluation Quality of Evidence Profile

| **Quality Assessment** | | | | | | **Summary of Findings** | | | | |
| --- | --- | --- | --- | --- | --- | --- | --- | --- | --- | --- |
| **Number of Studies*** | **Risk of Bias** | **Inconsistency** | **Indirectness** | **Imprecision** | **Publication Bias** | **Number of Participants** | | **Effect Size as SMD (95%** | **Significance** | **Quality** |
|  |  |  |  |  |  | **mHealth** | **Control** |  |  |  |
| Anxiety 5 (RCTs) | Very Serious 3/5 studies at high risk of bias based on Cochrane RoB 2 criteria. Blinding of participants and personnel was not feasible in most mHealth studies. | Very serious Very high level of heterogeneity (I² = 79%) with wide range of effect sizes across studies. | Not serious Populations and interventions are relevant to head and neck cancer rehabilitation. | Not serious Low baseline risk (<5%), narrow confidence interval, and moderate sample size (n = 250 per group) support precision. | Undetected Funnel plot asymmetry was not assessed due to limited number of studies; no evidence of publication bias from available data. | 262 | 242 | 0.75 (0.06, 1.44) | *p* = 0.03 | ●○○○ |
| Fatigue 6 (RCTs) | Very Serious 4/6 studies had high or some concerns for risk of bias, primarily due to inadequate blinding and incomplete outcome data. | Very serious Very high heterogeneity (I² = 79%) with substantial variation in effect sizes. | Not serious Fatigue is a common and well-defined outcome in HNC patients. Measurement tools are appropriate and widely used. | Not serious Sample size was adequate, and confidence interval is narrow, indicating sufficient precision. | Undetected No formal test for publication bias conducted due to small number of studies; funnel plot analysis not suggestive of systematic bias. | 378 | 391 | 0.69 (0.35, 1.04) | *p* < 0.001 | ●○○○ |
| QoL 12 (RCTs) | Very Serious 7/12 studies had high or some concerns for risk of bias, mainly due to lack of allocation concealment or high dropout rates. Blinding was not possible in most trials. | Very serious Very high heterogeneity (I² = 72%) with significant variation in outcomes. | Not serious Patient populations and interventions are directly relevant to post-treatment rehabilitation in HNC. | Not serious Large sample size, low baseline risk, and narrow confidence interval suggest good precision. | Undetected Funnel plot and eggers test (p = 0.593) do not suggest systematic publication bias. | 505 | 505 | 0.64 (0.61, 0.88) | *p* < 0.001 | ●○○○ |
| Pain 9 (RCTs) | Very Serious 5/9 studies had high or some concerns for risk of bias, including inadequate randomization and incomplete follow-up. Blinding was not feasi | Not serious Moderate heterogeneity (I2 = 36%), which is not statistically significant. Effect sizes were consistent across studies. | Not serious Pain is a key symptom in HNC patients, and measurement tools are standard and valid. | Not serious Sample size was adequate, and confidence interval is narrow, indicating sufficient precision. | Undetected No evidence of publication bias (eggers p = 0.784). | 385 | 385 | 0.37 (0.24, 0.49) | *p* < 0.001 | ●●○○ |
| Swallowing function 9 (RCTs) | Very Serious 5/9 studies had high or some concerns for risk of bias, particularly due to high attrition and missing data. Blinding was not possible. | Very serious Very high heterogeneity (I2= 91%) with wide range of effect sizes. | Not serious Swallowing dysfunction is highly relevant to HNC patients. Outcome measures are appropriate. | Not serious Sample size was moderate, and confidence interval includes zero but is narrow enough to suggest some benefit. | Undetected No formal test for publication bias; funnel plot analysis does not suggest systematic bias. | 430 | 428 | 0.46 (0.01, 0.90) | *p* = 0.04 | ●○○○ |
| Depression 5 (RCTs) | Very Serious 2/5 studies had high risk of bias (Chen 2021, Xu 2019) due to poor randomization and incomplete outcome data. Blinding was not feasible. | Very serious Very high heterogeneity (I2 = 83%) with substantial variation in effect sizes. Differences in depression scales (HADS, PHQ-9) and interven | Not serious Depression is a common comorbidity in HNC patients. Measurement tools are validated and widely used. | Not serious Sample size was adequate, and confidence interval is narrow, indicating sufficient precision. | Undetected No formal test for publication bias due to limited number of studies. | 262 | 262 | 0.88 (0.42, 1.35) | *p* < 0.001 | ●○○○ |
| MIO 4 (RCTs) | Very Serious 2/4 studies had high risk of bias due to inadequate randomization and high attrition. Blinding was not possible. | Very serious Very high heterogeneity (I2= 96%) with large variability in effect sizes. Differences in measurement protocols and timing of assessment m | Not serious MIO is a clinically relevant outcome in HNC rehabilitation. Measurement is objective and standardized. | Very serious Small sample size, wide confidence interval that includes zero, and non-significant result indicate serious imprecision | Undetected No formal test for publication bias; funnel plot analysis not applicable due to few studies. | 129 | 129 | 0.31 (-1.13, 1.75) | *p* = 0.68 | ●○○○ |

Abbreviations:

GRADE: Grading of Recommendations, Assessment, Development and Evaluation; RCT: Randomized Controlled Trial; CI: Confidence Interval; SMD: Standardized Mean Difference; MD: Mean Difference; RoB 2: Risk of Bias 2 tool; Number of studies that report results at end of intervention.

○○○○ Very low; ●●○○ Low; ●●●○ Moderate; ●●●● High

# Effect of mHealth Interventions for Patients With HNC

| **Outcomes** | **No. of study** | **I² value** | ***p*-value (Q)** | **Effects model** | **SMD (95% CI)** | **Prediction interval** | **p-value (Z)** |
| --- | --- | --- | --- | --- | --- | --- | --- |
| Anxiety | 5 | 93% | <0.001 | Random | -0.75 (-1.42; -0.08) | (-2.99; 1.49) | 0.03 |
| Fatigue | 6 | 78% | <0.001 | Random | -0.85 (-1.19; -0.51) | (-1.91; 0.21) | 0.00 |
| Quality of life | 12 | 72% | <0.001 | Random | 0.64 (0.41; 0.88) | (-0.18; 1.47) | 0.00 |
| Pain | 9 | 36% | 0.13 | Fixed | -0.37 (-0.49; -0.24) | NA | 0.00 |
| Swallowing function | 8 | 78% | <0.001 | Random | 0.66 (0.28; 1.04) | (-0.61; 1.93) | 0.04 |
| Depression | 5 | 84% | <0.001 | Random | -0.89 (-1.37; -0.40) | (-2.47; 0.70) | 0.00 |
| Maximal interincisal opening | 4 | 96% | <0.001 | Random | -0.37 (-2.80; 2.06) | (-9.12; 8.38) | 0.68 |

Abbreviations: CI, Confidence interval; NA, Not applicable.

# Characteristics and Functional Categories of mHealth Interventions

| **Characteristics** | | **Studies (N=29), n (%)** |
| --- | --- | --- |
| **Delivery Platforms** | | |
|  | Smartphone | 25 (86.2) |
|  | Tablet or Computer | 1 (3.4) |
|  | Smartwatch | 1 (3.4) |
|  | Web-based portal / Telemedicine system | 2 (6.9) |
|  | Telephone | 1 (3.4) |
| **Functional Modules** | | |
|  | Self-monitoring & reporting | 13 (44.8) |
|  | Home practice support | 14 (48.3) |
|  | Telemedical support | 13 (44.8) |
|  | Follow-up reminders | 17 (58.6) |
| **Intervention Format** | | |
|  | Interactive (Bi-directional communication) | 20 (69.0) |
|  | Didactic (Information-only / Unidirectional) | 9 (31.0) |

# Subgroup Analyses

| Outcome and Subgroup | No. of Studies | SMD (95% CI) | *P* value | I² (%) |
| --- | --- | --- | --- | --- |
| Anxiety | | | | |
| With Home Practice Support | 2 | 1.48 (1.12, 1.84) | <0.001 | 0 |
| Without Home Practice Support | 3 | 0.29 (0.06, 0.52) | 0.4 | 89 |
| With Telemedical Support | 3 | 0.55 (0.44, 0.66) | 0.007 | 85 |
| Without Telemedical Support | 2 | 1.06 (0.84, 1.28) | 0.03 | 85 |
| With Self-monitoring | 2 | 1.93 (1.07, 2.79) | 0.03 | 86 |
| Without Self-monitoring | 3 | 0.59 (0.45, 0.73) | 0.27 | 95 |
| Depression | | | | |
| With Home Practice Support | 2 | 1.53 (1.18, 1.88) | <0.001 | 0 |
| Without Home Practice Support | 3 | 0.49 (0.28, 0.70) | <0.001 | 83 |
| With Telemedical Support | 2 | 0.48 (0.19, 0.77) | 0.003 | 83 |
| Without Telemedical Support | 3 | 1.18 (0.89, 1.48) | <0.001 | 83 |
| With Self-monitoring | 2 | 0.97 (0.27, 1.67) | 0.07 | 89 |
| Without Self-monitoring | 3 | 0.84 (0.42, 1.26) | 0.009 | 87 |
| Swallowing Function | | | | |
| With Home Practice Support | 4 | 0.08 (-0.77, 0.93) | 0.85 | 94 |
| Without Home Practice Support | 5 | 0.74 (0.24, 1.23) | 0.004 | 87 |
| With Telemedical Support | 4 | -0.03 (-0.76, 0.71) | 0.95 | 93 |
| Without Telemedical Support | 5 | 0.83 (0.36, 1.30) | <0.001 | 84 |
| With Self-monitoring | 4 | 0.50 (0.31, 0.70) | <0.001 | 0 |
| Without Self-monitoring | 5 | 0.44 (-0.41, 1.29) | 0.31 | 95 |
| Short-term (≤3 months) | 2 | 0.59 (0.34, 0.85) | <0.001 | 0 |
| Long-term (>3 months) | 7 | 0.42 (-0.18, 1.02) | 0.17 | 93 |
| EORTC-based scales | 5 | 0.57 (0.40, 0.74) | <0.001 | 0 |
| MDADI | 2 | 1.25 (-0.56, 3.05) | 0.18 | 96 |
| Other scales | 2 | -0.54 (-2.05, 0.97) | 0.49 | 96 |
| Quality of Life | | | | |
| With Home Practice Support | 5 | 0.72 (0.19, 1.25) | 0.008 | 86 |
| Without Home Practice Support | 6 | 0.59 (0.39, 0.80) | <0.001 | 39 |
| With Telemedical Support | 5 | 0.59 (0.38, 0.81) | <0.001 | 33 |
| Without Telemedical Support | 6 | 0.70 (0.25, 1.16) | 0.002 | 84 |
| With Self-monitoring | 4 | 0.68 (0.36, 1.00) | <0.001 | 55 |
| Without Self-monitoring | 7 | 0.63 (0.30, 0.96) | <0.001 | 78 |
| Short-term (≤3 months) | 7 | 0.68 (0.38, 0.98) | <0.001 | 70 |
| Long-term (>3 months) | 4 | 0.74 (0.44, 1.03) | <0.001 | 52 |
| EORTC-based scales | 4 | 0.78 (0.52, 1.04) | <0.001 | 24 |
| Other scales | 7 | 0.58 (0.27, 0.90) | <0.001 | 78 |
| Fatigue | | | | |
| With Telemedical Support | 2 | 0.17 (0.04, 0.30) | 0.02 | 86 |
| Without Telemedical Support | 4 | 0.59 (0.17, 1.01) | 0.005 | 78 |
| With Self-monitoring | 2 | 0.32 (0.07, 0.57) | <0.001 | 83 |
| Without Self-monitoring | 6 | 0.65 (0.21, 1.09) | <0.001 | 79 |
| EORTC-based scales | 4 | 0.83 (0.49, 1.17) | 0.05 | 8 |
| Other scales | 2 | 0.69 (0.35, 1.03) | 0.04 | 97 |
| Pain | | | | |
| With Home Practice Support | 3 | 0.29 (0.06, 0.52) | 0.01 | 23 |
| Without Home Practice Support | 5 | 0.47 (0.25, 0.69) | <0.001 | 46 |
| With Telemedical Support | 4 | 0.34 (0.16, 0.52) | <0.001 | 62 |
| Without Telemedical Support | 4 | 0.39 (0.20, 0.58) | <0.001 | 10 |
| With Self-monitoring | 4 | 0.48 (0.24, 0.72) | <0.001 | 65 |
| Without Self-monitoring | 4 | 0.24 (0.06, 0.42) | 0.008 | 65 |
| Short-term (<3 months) | 3 | 0.38 (0.15, 0.61) | <0.001 | 55 |
| Long-term (>3 months) | 5 | 0.36 (0.18, 0.54) | <0.001 | 38 |
| EORTC-based scales | 6 | 0.51 (0.25, 0.77) | <0.001 | 0 |
| Other scales | 2 | 0.01 (-0.26, 0.28) | 0.94 | 94 |

# Sensitivity Analysis

| 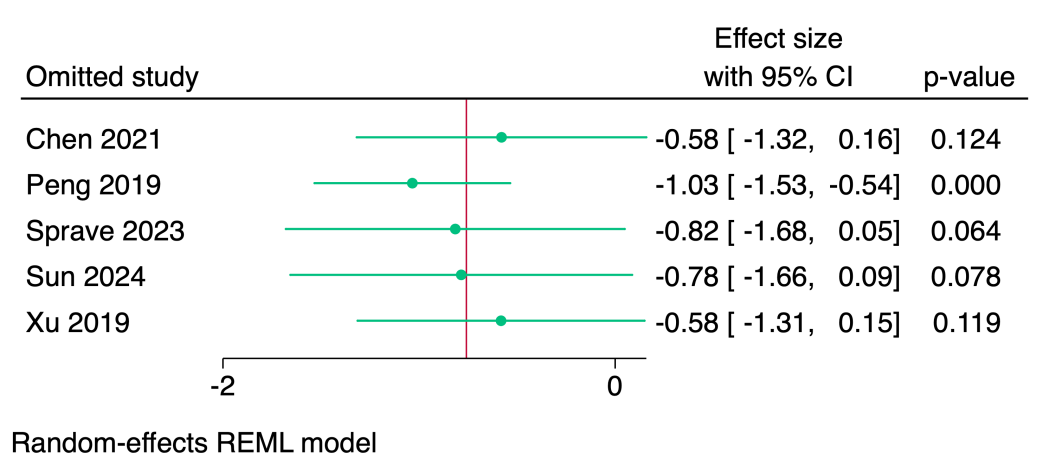  Figure S1. Sensitivity analysis of anxiety. | 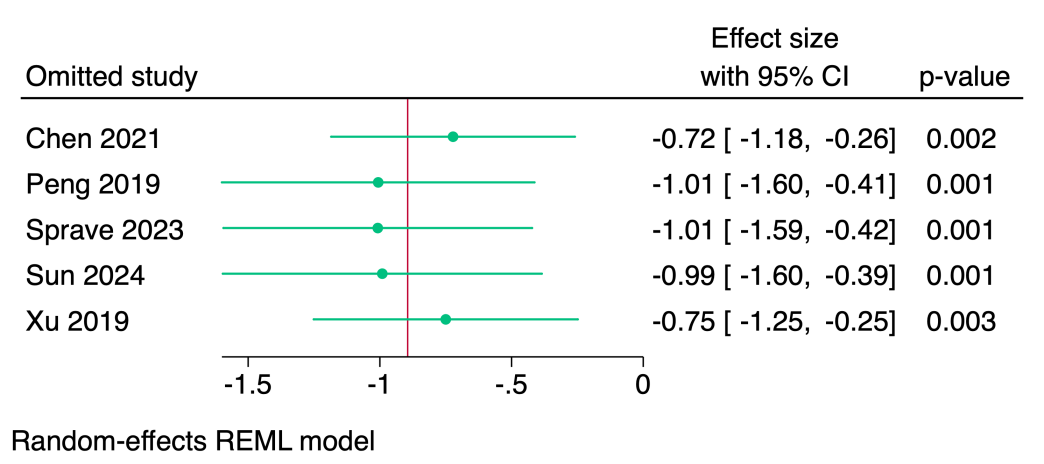  Figure S2. Sensitivity analysis of depression. |
| --- | --- |
| 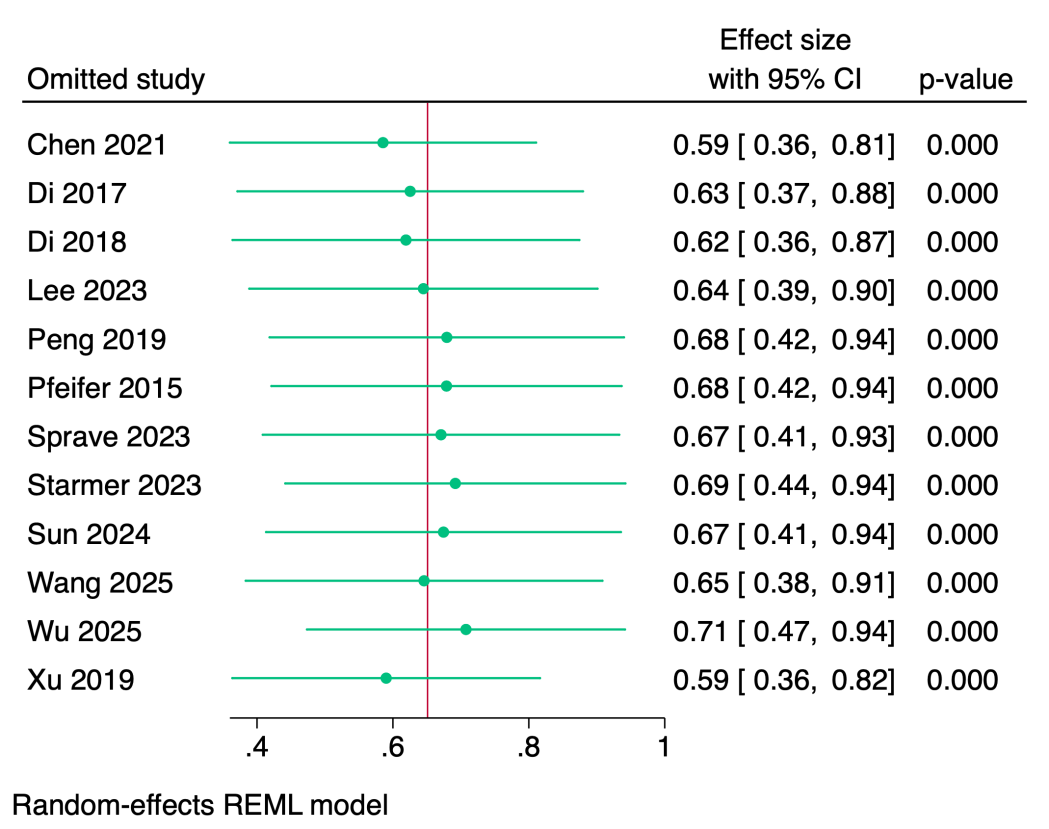  Figure S3. Sensitivity analysis on quality of life. | 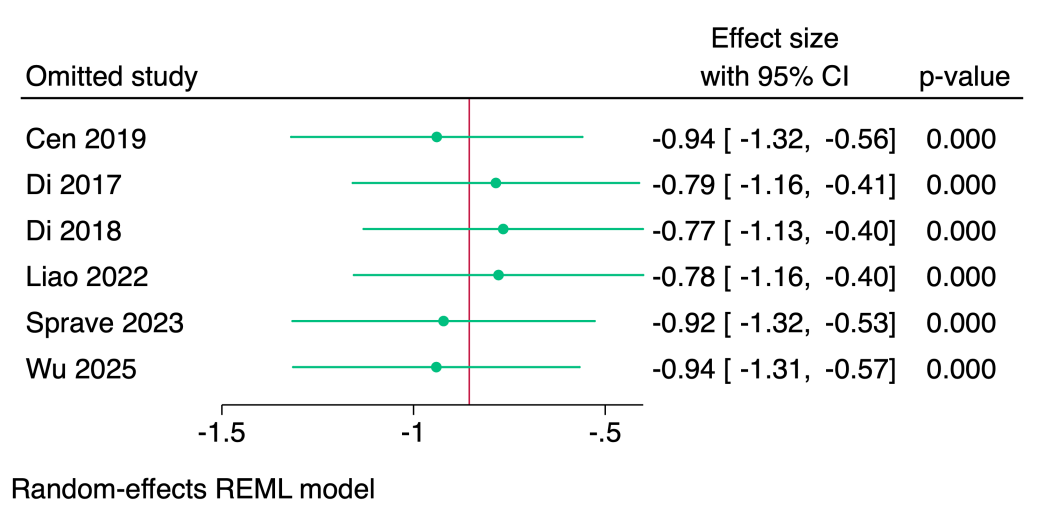  Figure S4. Sensitivity analysis of fatigue. |
| 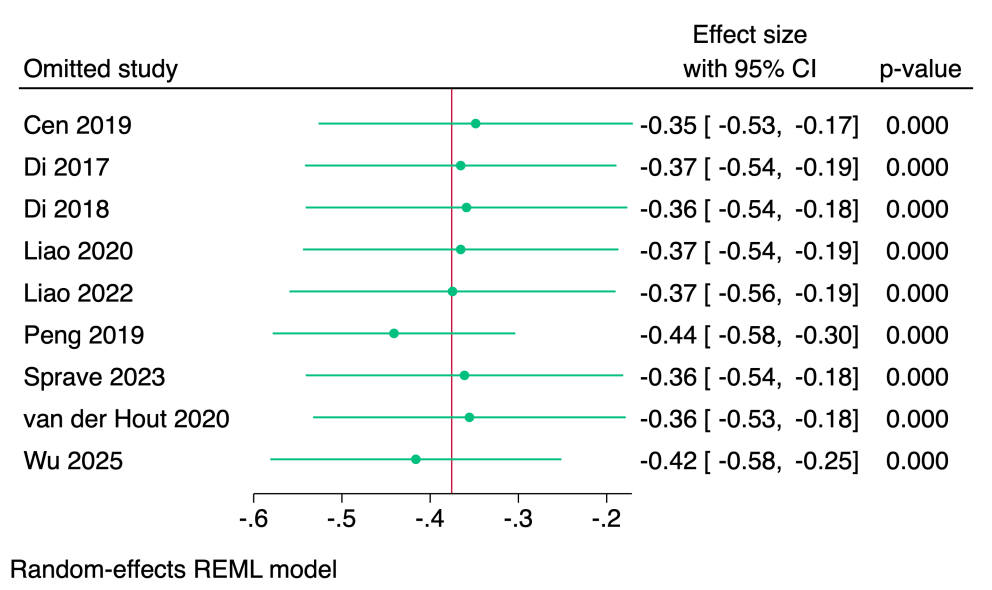  Figure S5. Sensitivity analysis of pain. | 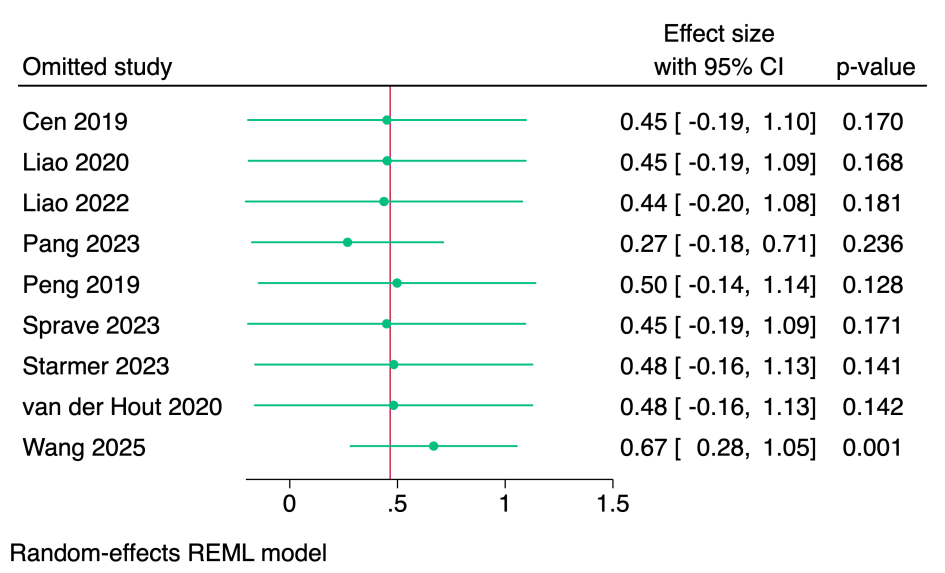  Figure S6. Sensitivity analysis of swallowing function. |
| 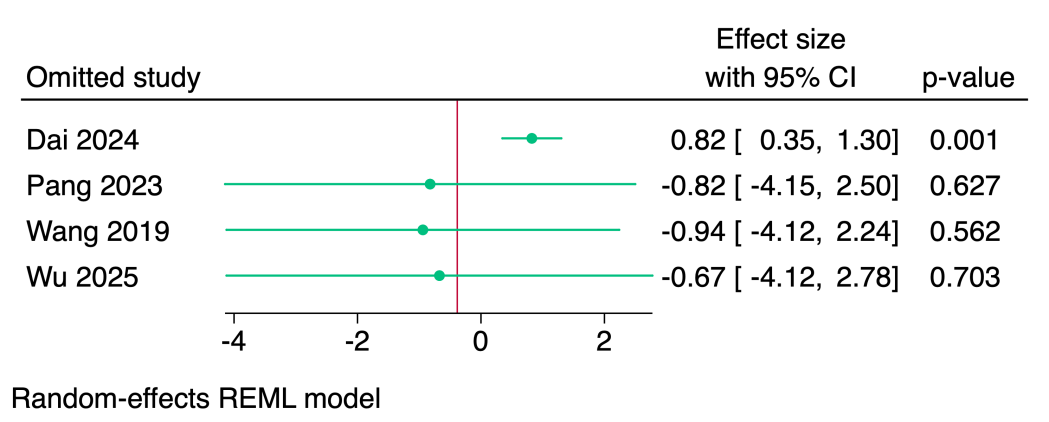  Figure S7. Sensitivity analysis of maximal interincisal opening. |  |

# Funnel Plots and Egger’s Tests

| 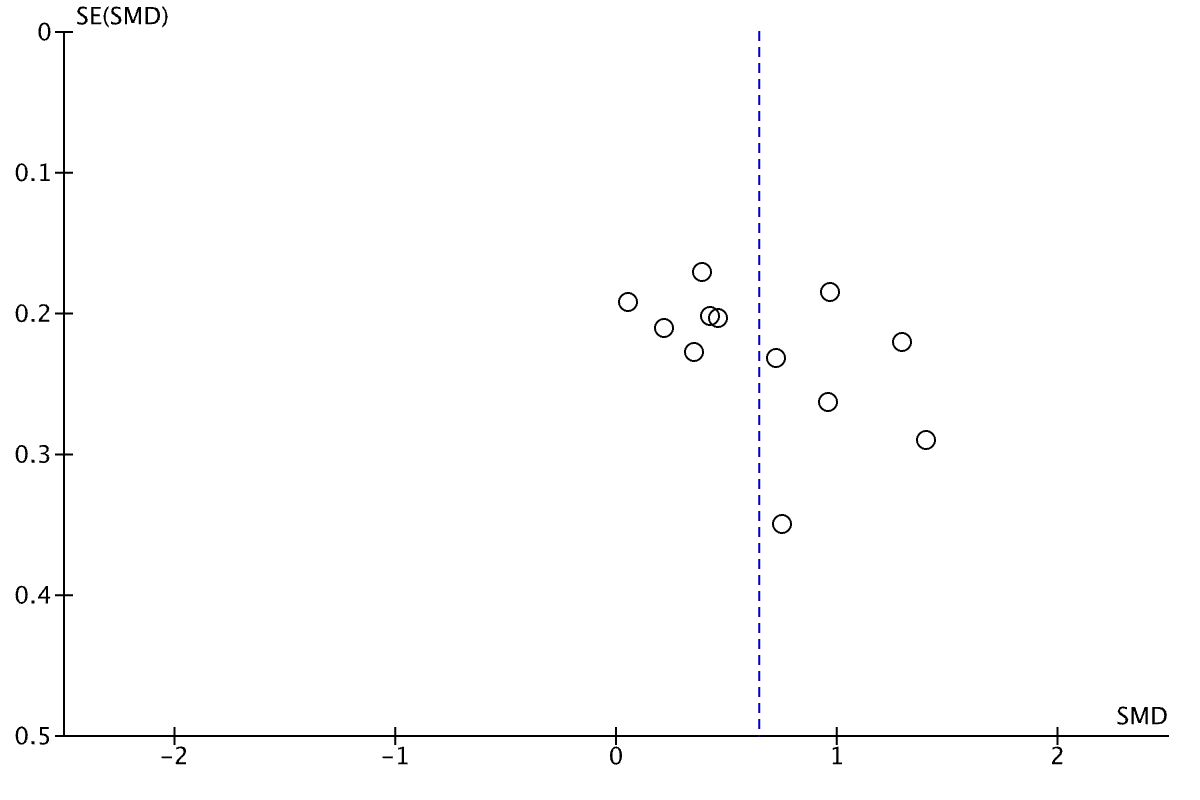  Figure S1. Funnel plot on quality of life. | 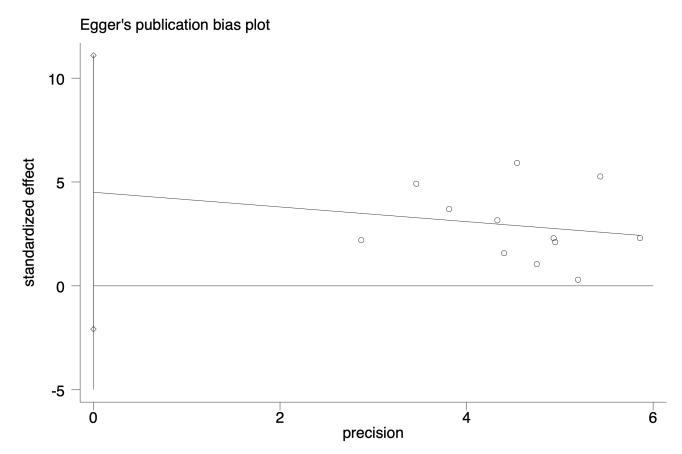  Figure S2. Egger test on quality of life. |
| --- | --- |
| 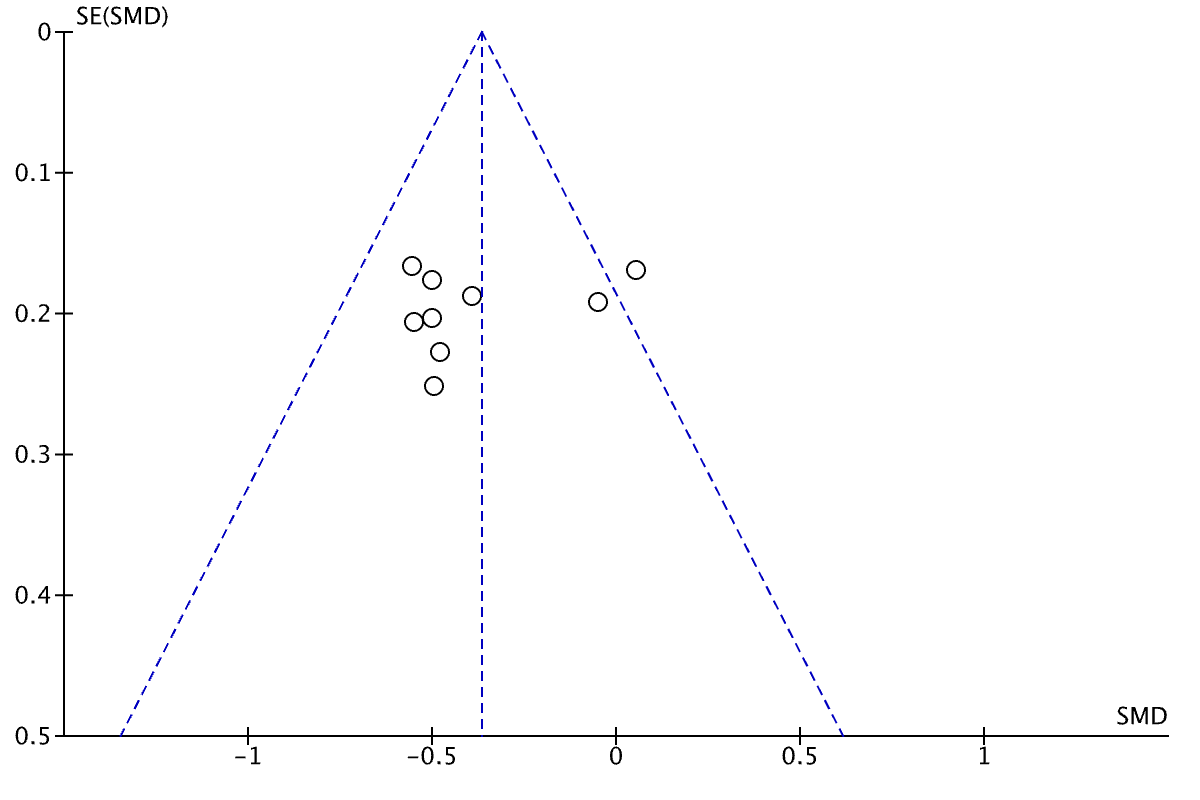  Figure S3. Funnel plot of pain. | 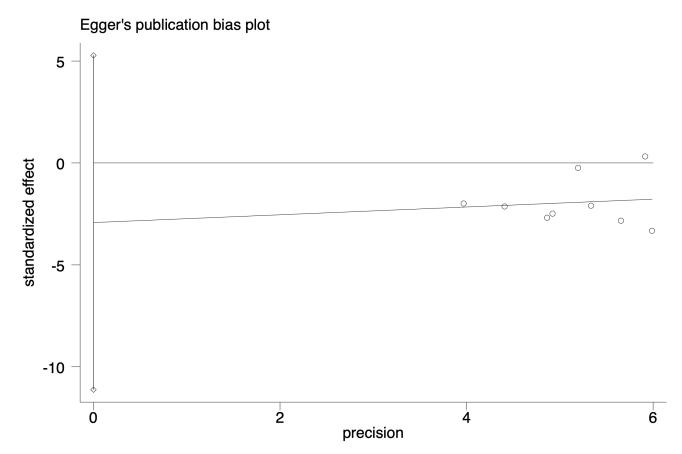  Figure S4. Egger test of pain. |
| 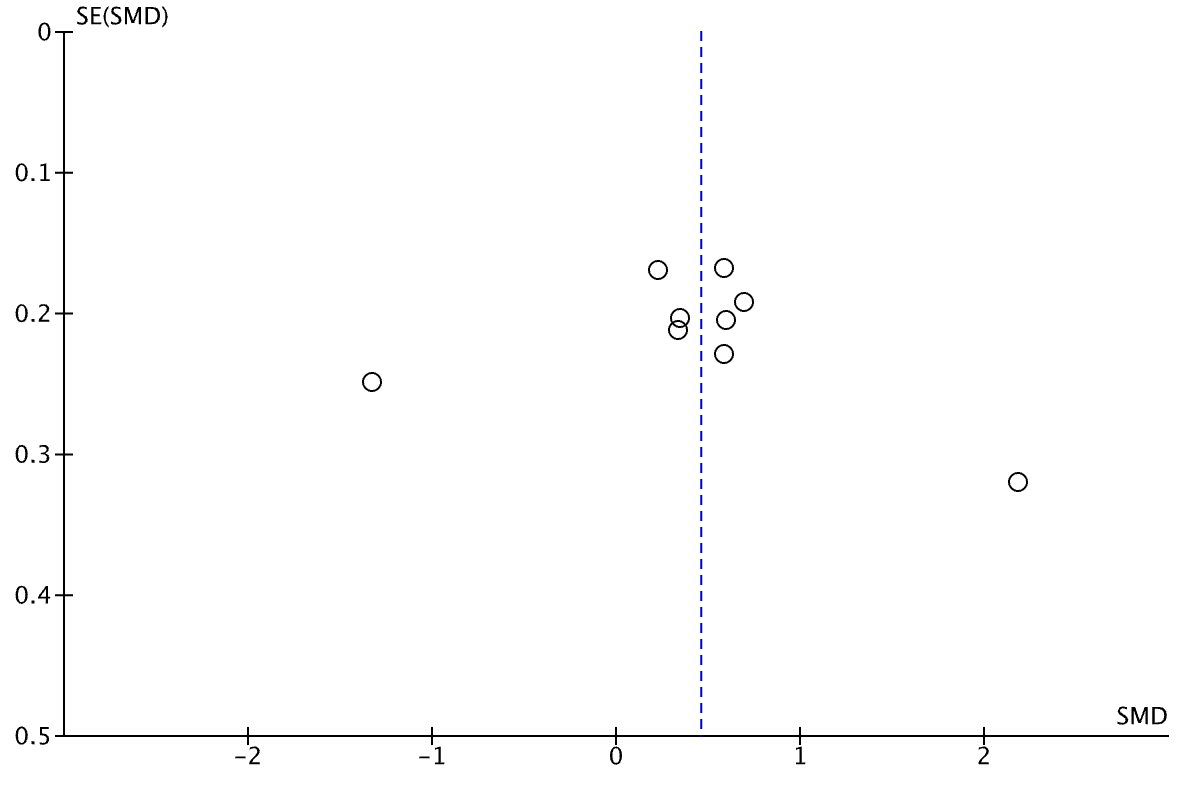  Figure S5. Funnel plot of swallowing function. | 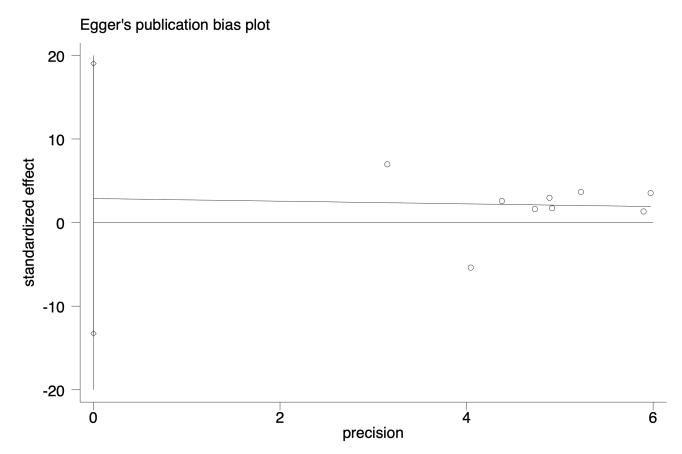  Figure S6. Egger test of swallowing function. |
